# Effectiveness of Virtual Reality–Complemented Pulmonary Rehabilitation on Lung Function, Exercise Capacity, Dyspnea, and Health Status in Chronic Obstructive Pulmonary Disease: Systematic Review and Meta-Analysis

**DOI:** 10.2196/64742

**Published:** 2025-04-07

**Authors:** Yuyin Chen, Yuanyuan Zhang, Xiuhong Long, Huiqiong Tu, Jibing Chen

**Affiliations:** 1 School of Nursing Guangxi University of Chinese Medicine Nanning China; 2 Department of Nursing Ruikang Hospital Affiliated to Guangxi University of Chinese Medicine Nanning China; 3 Center for Translational Medicine of Integrated Traditional Chinese and Western Medicine Ruikang Hospital Affiliated to Guangxi University of Chinese Medicine Nanning China

**Keywords:** virtual reality, video games, exergaming, pulmonary rehabilitation, chronic obstructive pulmonary disease, lung function, exercise capacity, dyspnea, health status, randomized controlled trial, systematic review, meta-analysis

## Abstract

**Background:**

Chronic obstructive pulmonary disease (COPD) is a progressive respiratory condition characterized by persistent airflow obstruction. Pulmonary rehabilitation (PR) is a cornerstone of COPD management but remains underutilized due to barriers such as low motivation and accessibility issues. Virtual reality (VR)–complemented PR offers a novel approach to overcoming these barriers by enhancing patient engagement and rehabilitation outcomes.

**Objective:**

This review aims to evaluate the effect of VR-complemented PR compared with comparators on lung function, exercise capacity, dyspnea, health status, and oxygenation in patients with COPD. Additionally, the study aimed to identify which comparator type (active exercise vs nonactive exercise control group) and intervention duration would result in the greatest improvements in rehabilitation outcomes. The study also assessed patient-reported experience measures, including acceptability and engagement.

**Methods:**

A comprehensive search of 11 international and Chinese databases identified randomized controlled trials (RCTs) published up to November 2024. Data were analyzed using RevMan 5.4, with pooled effect sizes reported as mean differences (MDs) and 95% CIs.

**Results:**

A total of 16 RCTs involving 1052 participants were included. VR-complemented PR significantly improved lung function (forced expiratory volume in 1 second [FEV1] [L], MD 0.25, *P*<.001; FEV1/forced vital capacity [FVC], MD 6.12, *P*<.001; FVC, MD 0.28, *P*<.001) compared with comparators. Exercise capacity, assessed by the 6MWD, significantly improved (MD 23.49, *P*<.001) compared with comparators; however, it did not reach the minimally clinically important difference of 26 m, indicating limited clinical significance despite statistical significance. VR-complemented PR also significantly reduced dyspnea measured by the modified British Medical Research Council scale (MD –0.28, *P*<.001), improved health status measured by the COPD Assessment Test (MD –2.95, *P*<.001), and enhanced oxygenation status measured by SpO2 (MD 1.35, *P*=.04) compared with comparators. Subgroup analyses revealed that VR-complemented PR had a significantly greater effect on FEV1 (L) (MD 0.32, *P*=.005) and 6MWD (MD 40.93, *P*<.001) compared with the nonactive exercise control group. Additionally, VR-complemented PR showed a greater improvement in FEV1/FVC (MD 6.15, *P*<.001) compared with the active exercise control group. Intervention duration influenced outcomes, with 5-12-week programs showing the greatest improvement in 6MWD (MD 38.96, *P*<.001). VR-complemented PR was well-accepted, with higher adherence and engagement rates than comparators.

**Conclusions:**

VR-complemented PR significantly improves lung function, exercise capacity, dyspnea, health status, and oxygenation in patients with COPD compared with comparators, while enhancing adherence and engagement. Subgroup analyses showed greater effects on FEV1 (L) and 6MWD compared with the nonactive exercise control group, and a larger improvement in FEV1/FVC compared with the active exercise control group. Interventions (5-12 weeks) yielded the most significant benefits in exercise capacity. These findings highlight VR as a promising adjunct to traditional PR, with future research focusing on long-term outcomes and standardized protocols.

## Introduction

Chronic obstructive pulmonary disease (COPD) is a prevalent and debilitating respiratory condition characterized by persistent symptoms and airflow obstruction [1]. Pulmonary rehabilitation (PR), a cornerstone of COPD management, has been widely shown to alleviate symptoms, improve functional capacity, and enhance health-related quality of life (HRQoL) [2,3]. Exercise training, a key component of PR, is particularly effective in increasing exercise capacity and reducing dyspnea [4,5]. However, despite its well-documented benefits, PR remains underutilized, with fewer than 5% of eligible patients accessing and completing these programs [6-8]. Barriers such as low motivation, transportation difficulties, psychological distress, and limited accessibility contribute to poor adherence and participation [9-11]. Therefore, innovative, patient-centered approaches are urgently needed to overcome these barriers and improve PR accessibility and effectiveness [12].

Virtual reality (VR) is a computer-generated simulation that creates immersive 3D environments, enabling interactive experiences through visual, auditory, tactile, and kinesthetic stimuli [13]. In PR, VR-enhanced rehabilitation improves patient motivation and engagement by offering customizable virtual settings, such as home environments or natural landscapes, with adaptable features such as intensity, duration, and real-time feedback [14,15]. For patients facing barriers to traditional PR, such as transportation difficulties or low motivation, VR provides an accessible, cost-effective, and flexible alternative, supporting rehabilitation even in home-based settings [16]. Extensive research has demonstrated the utility of VR in managing various conditions, including stroke [17], cancer [18], cerebral palsy [19], Parkinson disease [20], and spinal cord injury [21]. Studies show that VR enhances functional recovery, improves patient satisfaction, and promotes self-management, particularly in chronic disease populations. By increasing health care accessibility and empowering patients to take an active role in their rehabilitation, VR is a promising tool for advancing personalized and effective rehabilitation strategies.

Growing systematic review evidence suggests that VR-complemented PR may improve lung function, exercise capacity, dyspnea, and HRQoL in patients with COPD [22-26]. However, previous meta-analyses have notable limitations that warrant further investigation. For instance, Wang et al [22] included both randomized controlled trials (RCTs) and non-RCTs, but their quantitative analysis was limited to the 6-minute walk distance (6MWD), with dyspnea and HRQoL outcomes only descriptively summarized. Patsaki et al [23] and Obrero-Gaitán et al [26] restricted their analyses to English-language publications, potentially excluding relevant non-English studies. Chai et al [24] reported discrepancies in data presentation within the forest plots, raising concerns about the reliability of their findings. Furthermore, several reviews—including those by Patsaki et al [23], Chai et al [24], and Liu et al [25]—incorporated the study by Xie et al [27], which was later retracted due to quality concerns.

Given the growing number of trials on VR-complemented PR in COPD, an updated systematic evaluation was needed. This meta-analysis aimed to provide a high-quality synthesis of the evidence, assessing the impact of VR-complemented PR on lung function, exercise capacity, dyspnea, and health status as primary outcomes, while also evaluating secondary outcomes such as oxygenation status and patient-reported experience measures, including acceptability and engagement. Additionally, subgroup analyses were conducted based on factors such as comparator type (active group vs nonactive exercise control group) and intervention duration to identify conditions under which VR-complemented PR is most effective. By addressing these key outcomes with a rigorous methodological approach, this study aimed to offer a comprehensive and reliable understanding of the role and feasibility of VR-complemented PR in COPD rehabilitation.

## Methods

### Design

This systematic review and meta-analysis followed PRISMA (Preferred Reporting Items for Systematic Reviews and Meta-Analyses) guidelines [28]. The study protocol was registered with PROSPERO under registration number CRD42023472590.

### Information Sources and Search Strategy

A systematic literature search was conducted from the earliest available date to November 2024 across 11 databases, including 7 international sources (Web of Science, CINAHL, Cochrane Library, Scopus, PsycINFO, PubMed, and Embase) and 4 Chinese sources (SinoMed, CNKI, Wanfang, and VIP).

The search strategy included basic strings of Medical Subject Headings (MeSH) terms and free terms combined with Boolean operators. The search terms were “virtual reality,” “VR,” “virtual environment,” “video game*,” “virtual simulation,” “virtual medicine,” “mixed reality,” “commercial game*,” “virtual game*,” “exergam*,” “play-based therapy,” “augmented reality,” “virtual reality exposure therapy,” “x-box 360,” “Kinect,” “Wii,” “virtual world,” “head-mounted display,” “pulmonary disease, chronic obstructive,” “chronic obstructive pulmonary disease*,” “chronic obstructive airway disease,” “chronic obstructive lung disease,” “COAD,” “COPD,” “chronic airflow obstruction*,” “airflow obstruction, chronic,” and “airflow obstructions, chronic.” Multimedia Appendix 1 details the search strategies for each database. Our search included only Chinese and English sources and full-text articles from peer-reviewed journals. Additionally, we reviewed published reviews, reference lists of included studies, and similar articles. Before data synthesis, all databases were researched in December 2024 to capture newly published studies.

### Eligibility Criteria

The Participant, Intervention, Comparator, Outcomes, and Study Design (PICOS) model was used to establish the inclusion criteria for each article (Textbox 1).

**Inclusion and exclusion criteria.**

**1. Participants**

Adults (≥18 years) diagnosed with any stage of COPD according to the Global Initiative for Chronic Obstructive Lung Disease (GOLD) criteria were included.

**2. Interventions**

The intervention group in each study received VR-complemented PR. The VR component varied across studies and included features such as immersive environments, interactive exercises, and real-time feedback to create a motivating and engaging rehabilitation experience. PR was defined as a structured, comprehensive program including a combination of exercise training, respiratory training, and education aimed at improving physical and emotional well-being in patients with COPD. To qualify as PR, interventions required a minimum duration of 2 weeks and a frequency of at least two sessions per week. Studies with single-session or 1-day interventions were excluded. Although longer durations of 4-8 weeks are often recommended for PR programs in the literature [29,30], the inclusion of shorter-duration studies aligns with the GOLD report [31], which acknowledges that PR programs in many countries are frequently limited to less than 4 weeks due to resource constraints. Moreover, GOLD highlights the potential of VR-complemented PR as a viable alternative in such contexts [31].

**3. Comparator**

The comparator groups in the included studies were categorized based on the presence or absence of structured exercise interventions.

- Active exercise controls included structured and supervised exercise training interventions without VR components, aimed at improving physical fitness, lung function, and overall health. These interventions typically involved aerobic, resistance, endurance, strength, or respiratory muscle training. As part of comprehensive PR programs, they served as benchmarks to assess the additional benefits of VR-complemented PR.
- Nonactive exercise controls referred to interventions that did not include structured exercise training. Instead, they focused on standard COPD management, such as usual care, educational or behavioral interventions, or low-intensity PR programs without structured exercise (eg, breathing exercises, relaxation techniques, or daily activity guidance without specific training regimens). These comparators served as a reference to assess the overall effectiveness of VR-complemented PR compared with nonexercise-based approaches.

**4. Outcome measures**

Studies were included if they reported at least one primary outcome, such as lung function, exercise capacity, dyspnea, or health status. Secondary outcomes, such as oxygenation status and patient-reported experience measures (acceptability and engagement), were considered for qualitative synthesis. Outcomes with data from 2 or fewer studies were excluded from the meta-analysis due to concerns about statistical power and reliability, as limited data can lead to unreliable results and high variability.

**5. Study design**

Only RCTs were included. Quasi-experimental studies were excluded to ensure high-quality evidence for the review.

### Selection Process

Endnote X9 (Clarivate Plc) was used to export and manage all search results and to identify and remove duplicate studies. The screening process consisted of 2 stages. First, the titles and abstracts of the remaining articles were reviewed, and any study that examined the relationship between VR and COPD was retained for further analysis. After irrelevant articles were removed, the remaining articles were downloaded and further reviewed to determine which studies should be included in the final analysis. All steps were independently screened and cross-checked by 2 researchers (YC and YZ) against the eligibility criteria. Disagreements between the 2 reviewers were resolved through a consensus process involving additional investigators (HT and JC).

### Data Extraction

For each eligible study, a predesigned Excel form (Microsoft Corporation) was used for data extraction by an author (YC) on the following subheadings: (1) publication details (first author’s surname, publication year, and country); (2) participant characteristics (sample size, mean age, sex, and disease severity); (3) intervention details (site, exercise intensity, comparator group, intervention group, VR content, and intervention format); (4) outcome measures; and (5) both pre- and postintervention data (eg, mean and SD) for the intervention and comparator groups. For studies reporting SEs or median and IQR instead of means and SD, these values were converted using standard conversion tools [32,33]. The extracted data were reviewed by the second author (YZ) for accuracy.

### Quality Assessment

Two researchers (YC and XL) independently assessed the quality of the included studies. For RCTs, the Cochrane Risk of Bias tool [34] was used, which evaluates randomization sequence generation, allocation concealment, participant blinding, outcome blinding, incomplete outcome data, selective reporting, and other biases. Items were categorized as “low risk,” “high risk,” or “unclear risk.” Any discrepancies encountered during the review process were deliberated with other investigators (HT and JC) and ultimately reconciled through mutual agreement.

### Data Synthesis and Analysis

Statistical analysis was performed using Cochrane Review Manager 5.4 (Cochrane Collaboration) to assess the efficacy of VR-complemented PR in patients with COPD compared with comparators and to generate forest plots. Mean difference (MD) and 95% CI were calculated for continuous variables.

The *I*^2^ statistics were used to assess heterogeneity for each comparison. A fixed-effects model was applied when *P*0.10 and *I*^2^≤50%, indicating statistical homogeneity. Conversely, a random-effects model was used when heterogeneity was high (*P*<.10 and *I*^2^>50%).

Subgroup analyses were conducted to compare the efficacy of VR-complemented PR based on 2 factors: (1) comparator types (active exercise controls vs nonactive exercise controls) and (2) intervention duration (eg, ≤4 weeks, 5-12 weeks, and >12 weeks). These analyses aimed to explore differences in effectiveness across varying baseline conditions and intervention durations.

A sensitivity analysis was performed to assess the robustness of the findings by consecutively omitting each study. For the overall effect, a *P* value of less than .05 was considered statistically significant.

## Results

### Search Results and Selection

**Figure 1:**
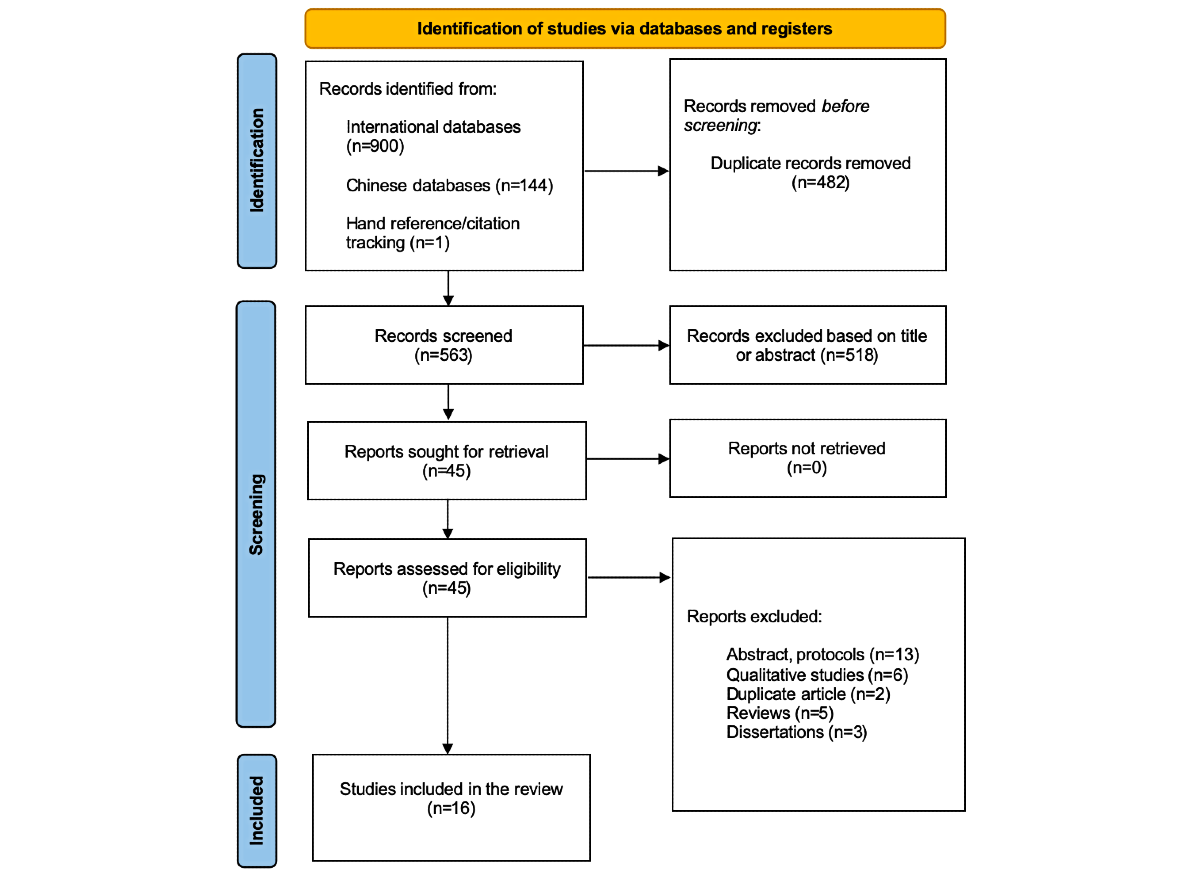
PRISMA flow diagram.

The search of 11 databases and other sources identified 1045 potentially relevant articles. After removing 482 duplicates and reviewing 563 titles and abstracts, 45 articles were selected for full-text screening. Finally, 16 articles were deemed eligible for inclusion in the meta-analysis. The literature screening process, reasons for exclusion, and results are illustrated in Figure 1 and Multimedia Appendix 2.

### Characteristics of the Included Studies

The features of the 16 trials [35-50] are displayed in Tables 1 and 2. The articles were published from 2014 to 2024, and 11 (69%) papers [40-50] were published within the last 5 years (2020-2024). In total, 9 (56%) [36,37,42-45,48-50] of the included studies were conducted in China, 3 (19%) in Poland [39-41], 2 (13%) in Italy [35,46], and 1 (6%) each in Indonesia [38] and Turkey [47].

**Table 1:** Publication details and characteristics of the participants.

Study	Country	Sample size	Age, mean (SD)	Sex (male/female), n	Disease severity (FEV_1_^a^, %pred or GOLD^b^), mean (SD)
Mazzoleni et al [35]	Italy	N=40 (IG^c^, n=20; CG^d^, n=20)	IG: 69 (11); CG: 74 (9)	Not reported	IG: 66.3 (19.3); CG: 59.7 (25.7)
Liu et al [36]	China	N=73 (IG, n=39; CG, n=34)	IG: 63 (9); CG: 63 (10)	40/33; IG: 22/17; CG: 18/16	Not reported
Hu et al [37]	China	N=60 (IG, n=30; CG, n=30)	IG: 74 (6); CG: 75 (5)	47/13; IG: 24/6; CG: 23/7	IG: 40.3 (8.6); CG: 42.4 (9.8)
Sutanto et al [38]	Indonesia	N=20 (IG, n=10; CG, n=10)	IG: 65 (8); CG: 66 (5)	19/1; IG: 9/1; CG: 10/0	IG: 49.1 (9.4); CG: 50.9 (13.1)
Rutkowski et al [39]	Poland	N=68 (IG, n=34; CG, n=34)	IG: 61 (4); CG: 62 (3)	35/33; IG: 17/17; CG: 18/16	IG: 62.9 (15.8); CG: 65.4 (24.0)
Rutkowski et al [40]	Poland	N=72 (IG, n=38; CG, n=34)	IG: 61 (4); CG: 62 (3)	37/35; IG: 19/19; CG: 18/16	IG: 60.5 (16.2); CG: 65.4 (24.0)
Rutkowski et al [41]	Poland	N=50 (IG, n=25; CG, n=25)	IG: 64 (6); CG: 68 (9)	9/41; IG: 4/21; CG: 5/20	IG: 71.0 (23.7); CG: 86.5 (21.1)
Zhou et al [42]	China	N=119 (IG, n=61; CG, n=58)	IG: 71 (7); CG: 71 (6)	94/25; IG: 48/13; CG: 46/12	GOLD stages Ⅱ-Ⅳ
Liu et al [43]	China	N=100 (IG, n=50; CG, n=50)	IG: male 74, female 76; CG: male 75, female 75	78/22; IG: 38/12; CG: 40/10	IG: 40.3 (10.7); CG: 39.2 (8.6)
Zhu et al [44]	China	N=43 (IG, n=22; CG, n=21)	IG: 65 (13); CG: 65 (13)	29/14; IG: 15/7; CG: 14/7	IG: GOLD Ⅱ=8, GOLD Ⅲ=10, GOLD Ⅳ=4; CG: GOLD Ⅱ=9, GOLD Ⅲ=8, GOLD Ⅳ=4
Xu [45]	China	N=70 (IG, n=35; CG, n=35)	IG: 66 (3); CG: 66 (3)	37/33; IG: 17/18; CG: 20/15	Not reported
Pancini et al [46]	Italy	N=17 (IG, n=8; CG, n=9)	IG: 72 (9); CG: 73 (8)	10/7; IG: 4/4; CG: 6/3	IG: GOLD I=2, GOLD Ⅱ=3, GOLD Ⅲ=3; CG: GOLD I=2, GOLD Ⅱ=3, GOLD Ⅲ=4
Kizmaz et al [47]	Turkey	N=50 (IG, n=25; CG, n=25)	IG: 63 (7); CG: 64 (7)	49/1; IG: 25/0; CG: 24/1	IG: GOLD Ⅱ=3, GOLD Ⅲ=14, GOLD Ⅳ=8; CG: GOLD Ⅱ=5, GOLD Ⅲ=13, GOLD Ⅳ=7
Wang et al [48]	China	N=80 (IG, n=40; CG, n=40)	IG: 72 (3); CG: 72 (3)	44/36; IG: 23/17; CG: 21/19	Not reported
Wang et al [49]	China	N=68 (IG, n=34; CG, n=34)	IG: 56 (8); CG: 56 (8)	40/28; IG: 21/13; CG: 19/15	IG: GOLD Ⅱ=6, GOLD Ⅲ=19, GOLD Ⅳ=9; CG: GOLD Ⅱ=7, GOLD Ⅲ=24, GOLD Ⅳ=3
Wei et al [50]	China	N=122 (IG, n=61; CG, n=61)	IG: 72 (6); CG: 72 (6)	70/52; IG: 35/26; CG: 35/26	Not reported

^a^FEV_1_%: percent predicted normal values of FEV_1_.

^b^GOLD: Global Initiative for Chronic Obstructive Lung Disease guidelines: GOLD I (FEV_1_≥80%), GOLD Ⅱ (50%≤FEV_1_ < 80%), GOLD Ⅲ (30%≤FEV_1_<50%), and GOLD Ⅳ (FEV_1_<30%).

^c^IG: intervention group.

^d^CG: comparator group.

**Table 2:** Characteristics of interventions and outcomes.

Study	Site	Comparator group	Intervention group	VR^a^ content	Intervention format	Exercise intensity	Outcome
Mazzoleni et al [35]	Hospital (inpatient)	PR^b^ (optimization of drug therapy, incremental treadmill, cycle, and arm ergometer exercises; abdominal, upper, and lower limb muscle activities; education; nutritional programs; and psychosocial counseling)	PR + VR sessions of Wii Fit Plus exercises	Wii Fit Plus: includes activities such as yoga, jogging plus, and twisting and squat	Length: 60 minutes/session; frequency: 1/day; duration: 3 weeks (7 days in the final week for the intervention group)	PR exercise: Borg dyspnea scale 5; VR exercise: Borg Dyspnea Scale 4-6	6MWT^c^, mMRC^d^, acceptability
Liu et al [36]	Hospital (inpatient and outpatient)	Usual care (medication therapy) + PR (health education, respiratory muscle training, and traditional upper and lower limb exercises)	Usual care + PR + VR-assisted upper and lower limb exercises using the BioMaster VR system	Upper limb: simulated activities such as board wiping, tea serving, and soup pouring; lower limb: simulated cycling exercise	Length: 15-40 minutes/session; frequency: 5 times/week; duration: 20 weeks	Not reported	FEV_1_^e^ (L), FEV_1_/FVC^f^, 6MWT, CAT^g^
Hu et al [37]	Hospital (inpatient and outpatient)	Usual care (medication therapy) + PR (respiratory muscle training, COPD^h^ education, smoking cessation, nutritional guidance, and traditional upper and lower limb exercises)	Usual care + PR + VR-assisted upper and lower limb exercises using the BioMaster VR system	Upper limb: simulated activities such as household chores and kitchen tasks; lower limb: simulated cycling exercise	Length: 10-30 minutes/session; frequency: 5 times/week; duration: 12 weeks	Not reported	FEV_1_ (%), FEV_1_/FVC, 6MWT, CAT
Sutanto et al [38]	Hospital (outpatient)	Exercise training (cycle ergometer sessions)	Exercise training + video-game assisted program by Wii Fit	Wii Fit program: includes yoga (deep breathing and poses), torso twist (strength training), and free run (aerobic exercise)	Length: 30 minutes/session; frequency: 3 times/week; duration: 6 weeks	Exercise training: Borg Dyspnea Scale 5; and VR exercise: adjusted to tolerance, monitored by heart rate, SpO_2_, and respiratory rate	6MWT and mMRC
Rutkowski et al [39]	Not reported	PR (physical capacity training, breathing exercises, physical exercise, inspiratory muscle training, inhalations, and relaxation)	PR + VR exercise training using Xbox 360 and Kinect motion sensor	Kinect Adventures mini-games: Rafting (paddling movements), Cross-Country Running, Hitting a Ball, and Roller-Coaster Ride	Length: not reported; frequency: 1/day; duration: 14 days (2 weeks)	Not reported	6MWT
Rutkowski et al [40]	Hospital (inpatient)	Exercise training (stationary cycle ergometer exercise) + PR (fitness, respiratory exercises, group walks, inhalation therapy, postural drainage, chest percussion, and relaxation training)	Exercise training + PR + VR exercise training using Xbox 360 and Kinect motion sensor	Kinect Adventures mini-games: 20,000 Leaks, Curvy Creek, Rally Ball, Reflex Ridge	Length: 20 minutes/session; frequency: 5 times/week; duration: 2 weeks	Endurance exercise: 60%-70% of max HR^i^ (based on 6MWT), 70% for GOLD^j^ 2, and 60% for GOLD 3; VR exercise: HR monitored to stay below age-predicted max (208 – 0.7 × age)	6MWT
Rutkowski et al [41]	Hospital (inpatient)	PR (fitness, diaphragm strengthening, exhalation, chest percussion, inhalation, cycle ergometer exercise, and 10 sessions of Schultz autogenic relaxation training)	PR + VR therapy with VR TierOne device	Virtual Therapeutic Garden (TierOne device; Stolgraf), based on Ericksonian psychotherapy, aimed at emotional balance and recovery	Length: 20 minutes/session; frequency: 5 times/week; duration: 2 weeks	Training heart rate based on GOLD spirometric stages	6MWT and FEV_1_ (%)
Zhou et al [42]	Home	PR (health education, diet, exercise, breathing techniques, breathing exercises, upper and lower limb strength exercises, and one-on-one guidance by respiratory nurses)	PR + VR training by Kinect motion sensor	The VR games include Cross-Country Running, Rafting, Ball Hitting, and Mountain Bike Simulation	Length: 30 minutes/session; frequency: 5 times/week; duration: 24 weeks	Not reported	FEV_1_ (%), FEV_1_/FVC, SpO_2_^k^, 6MWT, mMRC, and adherence rate
Liu et al [43]	Hospital (inpatient and outpatient)	Usual care (sputum removal, bronchiectasis treatment, and inhaled glucocorticoids) + PR (health education, smoking cessation, nutritional guidance, respiratory muscle training, and upper and lower limb training)	Usual Care + PR + VR training by BioMaster virtual scene interactive rehabilitation training system	Cycling simulation was selected for lower limb training	Length: 5-15 minutes/session; frequency: 1 time/day; duration: 12 weeks	Not reported	FEV_1_ (%), 6MWT, and CAT
Zhu et al [44]	Not reported	PR (health education, nutritional guidance, breathing exercises, aerobic and resistance training, and regular follow-up)	PR + interactive body-sensing VR training using Kinect 2.0, Xbox 360, and Nuts E9i projector	The VR games included Cross-Country Running, Fruit Ninja, and Obstacle Skiing	Length: 15-35 minutes/day (increased by 10 minutes/week); frequency: 1 time/day, 5 times/week; duration: 16 weeks	Not reported	FEV_1_ (L), FEV_1_/FVC, FVC, 6MWT, mMRC, CAT, and adherence rate
Xu [45]	Not reported	Usual care (monitoring vital signs and medications, basic nursing [eg, turning, sputum suction, oral care, nasogastric nutrition], regular respiratory tubing replacement and disinfection, and rehabilitation education on COPD pathology, treatment methods, lifestyle changes, and dietary guidance)	Usual care + PR + VR training + exercise training (comprehensive respiratory training involving pursed-lip breathing and diaphragmatic breathing)	Virtual household activities (eg, cleaning, organizing) with exercises such as box lifting (5 minutes for upper limbs) and gait training (10 minutes for lower limbs)	Length: 15 minutes/session; frequency: 5 times/week; duration: 12 weeks	Not reported	FEV_1_ (L), FEV_1_/FVC, FVC, SpO_2_, 6MWT, CAT, and satisfaction
Pancini et al [46]	Hospital (inpatient)	PR + relaxing music listening	PR + VR-based intervention	Relaxing virtual scenario with the Oculus Quest 2 headset and narrative voice, followed by a savoring exercise via prerecorded audio	Length: 25 minutes/session; frequency: 2 sessions/week; duration: 2 weeks	Not reported	SpO_2_
Kizmaz et al [47]	Hospital (inpatient)	Usual care (medication therapy) + PR (respiratory control, diaphragmatic breathing, thoracic expansion exercises, pursed-lip breathing, dyspnea reduction positions, relaxation exercises, cough/huffing training, upper extremity exercises, and walking exercises)	Usual care (medication therapy) + PR + VR cycling simulation using the Oculus Quest 2 headset	The Oculus Quest 2 headset was used to create a virtual cycling simulation in a forest, utilizing 360-degree real-world footage for ecological realism, rather than active video games	Length: based on patient tolerance; frequency: 1 time/day, 5 times/week; duration: ongoing until discharge	Target HR: heart rate [(max HR – resting HR) × (40 or 60)%] + resting HR	mMRC and CAT
Wang et al [48]	Not reported	Usual care (education, lifestyle modification, psychological support, dietary guidance, light physical activity [suggested only, no structured plan], and regular follow-up calls)	VR training by BioMaster + exercise training (multidimensional breathing training: deep breathing, pursed-lip breathing, diaphragmatic breathing, candle-blowing, seated breathing, and stair climbing)	Simulated virtual scenarios: household activities (cooking), cycling, real-time feedback, and adjustment	Length: 5-15 minutes/day; frequency: 1 time/day; duration: 12 weeks	Not reported	FEV_1_ (L), FEV_1_/FVC, and FVC
Wang et al [49]	Not reported	Usual care	VR-assisted cognitive behavioral nursing + exercise training (breathing exercises: abdominal and mindful breathing)	Virtual park exposure (walking simulation), disease knowledge education videos, and breathing exercises (abdominal and mindful breathing)	Length: 30-40 minutes/session; frequency: 2 times/week; duration: 8 weeks	Paused if heart rate > (207 − 0.7 × age) bpm or SpO_2_ < 88%, with rest or oxygen supplementation	6MWT
Wei et al [50]	Hospital (inpatient) and home	Usual care (medication therapy, oxygen therapy, positioning guidance, and health education) + PR (breathing exercises, nutritional support, self-care education, disease knowledge, and other standard discharge instructions)	Usual care + PR + VR training using the SUBOR A20 gaming console	The VR games included Ping-Pong Master, Swimming Master, and Kitchen Knife Master	Length: 20 minutes/session; frequency: not reported; duration: 8 weeks	Not reported	6MWT and mMRC

^a^VR: virtual reality.

^b^PR: pulmonary rehabilitation.

^c^6MWT: 6-minute walk distance.

^d^mMRC: modified British Medical Research Council.

^e^FEV_1_: forced expiratory volume in 1 second.

^f^FVC: forced vital capacity.

^g^CAT: COPD Assessment Test.

^h^COPD: chronic obstructive pulmonary disease.

^i^HR: heart rate.

^j^GOLD: Global Initiative for Chronic Obstructive Lung Disease.

^k^SpO_2_: peripheral capillary oxygen saturation.

### Study Participants

The trials recruited a total of 1052 people with COPD. Fifteen studies [36-50] reported the sex of the participants (1012 individuals), with 638 (63.04%) women and 374 (36.96%) men. Additionally, 1 study [35] did not provide information on sex. Twelve studies [35,37-44,46,47,49] provided data on the severity of COPD in the participants. According to the GOLD criteria, COPD severity was classified as stage I to III in 1 study [46] and as stage II to IV in 4 studies [42,44,47,49]. Additionally, 7 studies [35,37-41,43] reported the percentage of predicted forced expiratory volume in 1 second (FEV_1_% pred), with values ranging from 39.2% to 86.5%. Based on both the GOLD stages and FEV_1_% pred, the majority of patients in this review had COPD severity ranging from moderate to very severe.

### Intervention

A total of 6 studies [35,38,40,41,47,49] reported exercise intensity. Among them, 5 studies [38,40,41,47,49] monitored heart rate during exercise to ensure the intensity remained within the target range, while 1 study [35] assessed exercise intensity using the Borg dyspnea scale either during or immediately after the exercise session. Of the included studies, 11 [35-44,47] were categorized as active exercise controls with structured and supervised exercise training, while 5 [45,46,48-50] were classified as nonactive exercise controls, focusing on standard COPD management or low-intensity, nonstructured interventions. The VR technologies used in the studies varied, with 14 studies providing information on the types of VR technologies used: 2 (14%) [35,38] studies used the Nintendo Wii, 4 (29%) [39,40,42,44] used the Microsoft Xbox Kinect, 4 (29%) [36,37,43,48] used the BioMaster system, 2 (14%) [46,47] used the Oculus Quest 2, 1 (7%) [41] used the TierOne device (Stolgraf), and 1 (7%) [50] used the SUBOR A20 gaming console (Xiaobawang Company). Additionally, 2 studies [45,49] did not report the type of VR technology used. Interventions ranged from 2 weeks to 24 weeks, with frequency occurring 2-7 days per week. The duration of each intervention varied from 5 minutes to 1 hour.

### Outcome Measures

Lung function was assessed using FEV_1_ (%), FEV_1_ (L), forced vital capacity (FVC), and FEV_1_/FVC (%). Exercise capacity was evaluated using the 6MWD test. Dyspnea severity was measured using the modified British Medical Research Council (mMRC) scale. Health status was assessed with the COPD Assessment Test (CAT). Secondary outcomes included oxygenation status, assessed by peripheral capillary oxygen saturation (SpO_2_), and patient-reported experience measures such as acceptability and engagement.

### Risk of Bias Assessment

**Figure 2:**
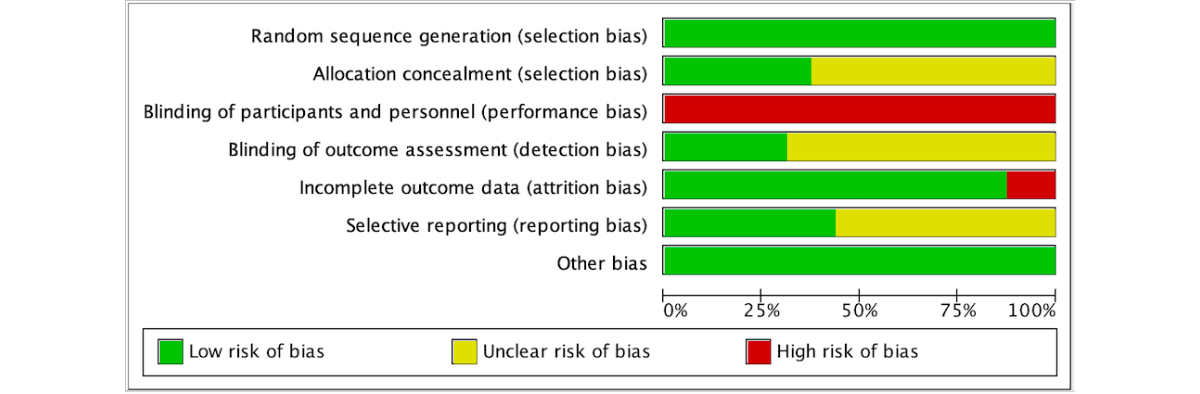
Cochrane risk of bias graph for randomized controlled trials.

**Figure 3:**
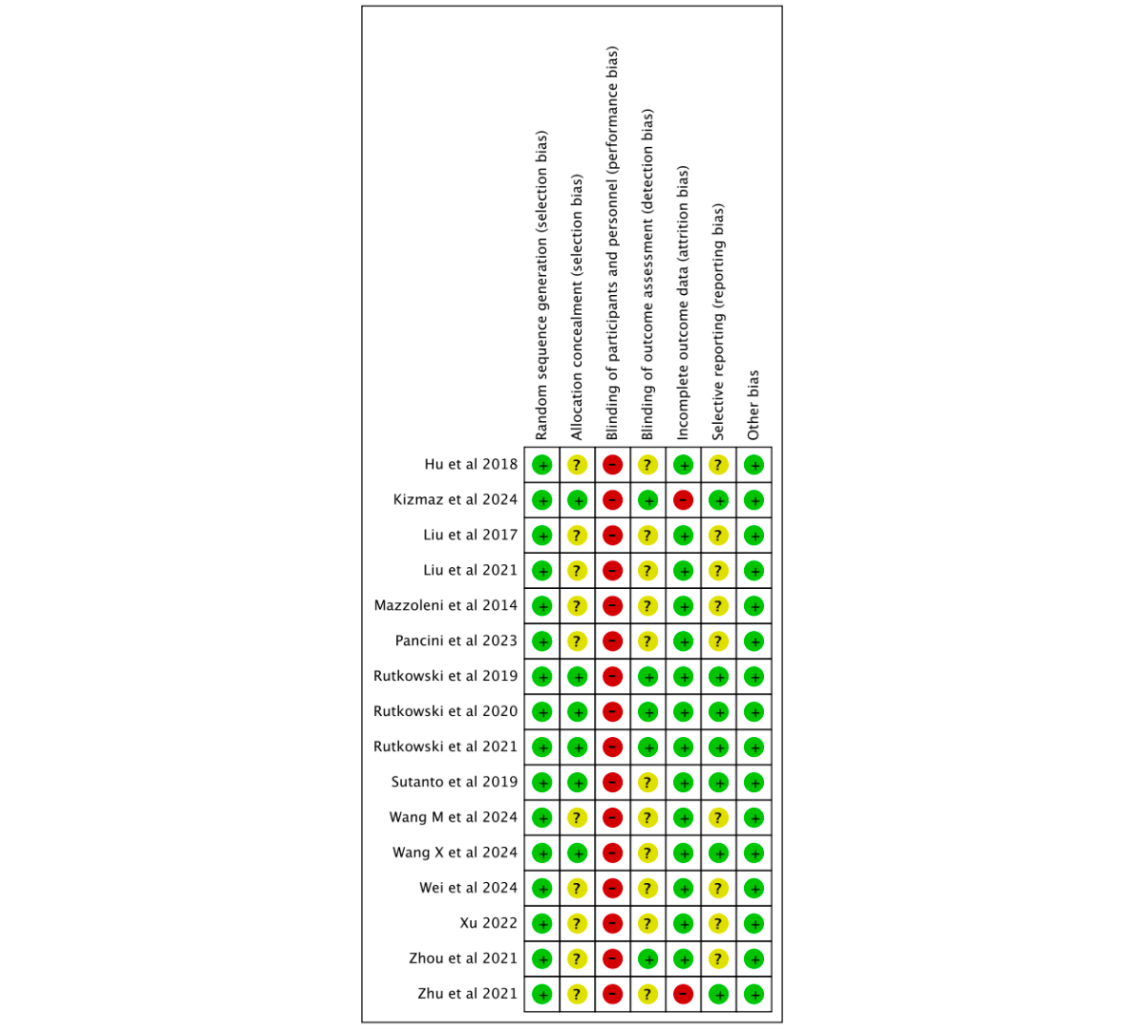
Cochrane risk of bias summary for randomized controlled trials.

Figures 2 and 3 (see also [35-50]) illustrate the results of the Cochrane Risk of Bias Tool, which was applied to evaluate the quality of the RCTs included in this analysis. All 16 RCTs reported random sequence generation, and 6 (38%) studies provided details on allocation concealment. Because of the nature of the VR intervention, none of the studies were able to blind participants or personnel. However, 5 (31%) studies implemented blinding of outcome assessors, which reduced the risk of detection bias. A total of 14 (88%) studies reported complete outcome data, while 7 (44%) offered sufficient information to evaluate the risk of selective reporting. Additionally, all studies were considered to have a low risk of other biases.

### Meta-Analysis of Outcomes

#### Lung Function

##### FEV1 (L)

**Figure 4:**
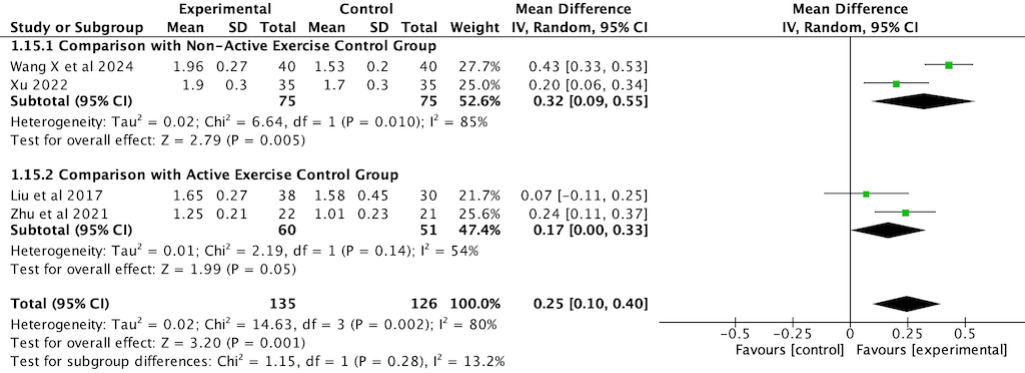
Forest plot of FEV1 (L) to assess lung function. FEV1: forced expiratory volume in 1 second.

Four RCTs [36,44,45,48] measuring FEV_1_ (L) were pooled for meta-analysis, involving a total of 261 participants. Subgroup analysis based on comparator type showed that VR-complemented PR had a significantly greater effect on FEV_1_ (L) compared with nonactive exercise controls (MD 0.32, 95% CI 0.09-0.55, *P*=.005), but with high heterogeneity (*I*^2^=85%). By contrast, the effect compared with active exercise controls was smaller (MD 0.17, 95% CI 0.00 to 0.33, *P*=.05), with moderate heterogeneity (*I*^2^=54%). Given the high heterogeneity, a random-effects model was applied. The overall results suggest that VR-complemented PR significantly improves FEV_1_ (L) compared with the comparators, with a pooled effect size of 0.25 (95% CI 0.10-0.40, *P*=.001), although moderate heterogeneity was observed across all studies (*I*^2^=80%, *P*=.002; Figure 4; see also [36,44,45,48]). Variations in measurement time, methods, and intervention protocols may have contributed to the high heterogeneity observed.

**Figure 5:**
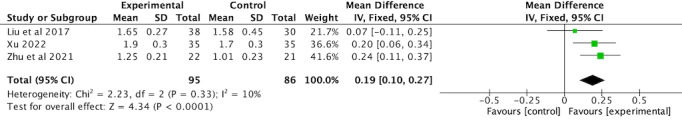
Forest plot of FEV1 (L) for lung function assessment after sensitivity analysis. FEV1: forced expiratory volume in 1 second.

To address the high heterogeneity observed in the analysis, a sensitivity analysis was performed by excluding the study by Wang et al [48]. This study was excluded due to its larger sample size compared with the other studies, which may have contributed to an overrepresentation of the study’s effect size and increased heterogeneity. After the removal of this study, heterogeneity decreased to 10% (*I*^2^=10%, *P*=.33), and a fixed-effect model was applied. The revised analysis showed that the improvement in FEV_1_ (L) remained significant (MD 0.19, 95% CI 0.10-0.27, *P*<.001; Figure 5; see also 36,45,44).

##### FEV1 (%)

**Figure 6:**
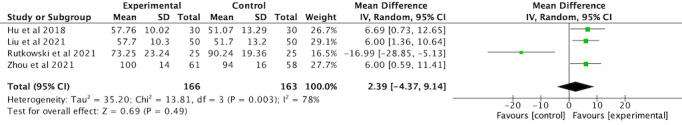
Forest plot of FEV1 (%) to assess lung function. FEV1: forced expiratory volume in 1 second.

Four RCTs [37,41-43] involving a total of 329 participants with COPD evaluated the effects of VR-complemented PR on FEV_1_ (%). The heterogeneity test indicated significant variability among the studies (*I*^2^=78%, *P*=.003), likely due to differences in study protocols, participant characteristics, and the intensity of the interventions. A random-effects model was applied because of the high heterogeneity across the studies. The meta-analysis showed no statistically significant difference in FEV_1_ (%) between the intervention and comparator groups (MD 2.39, 95% CI –4.37 to 9.14, *P*=.49; Figure 6; see also [37,41-43]).

**Figure 7:**
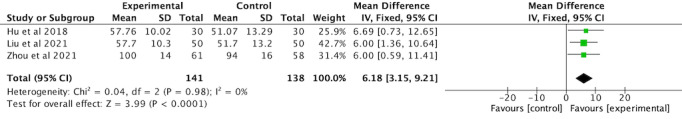
Forest plot of FEV1 (%) for lung function assessment after sensitivity analysis. FEV1: forced expiratory volume in 1 second; FVC: forced vital capacity.

Because of the high heterogeneity, a sensitivity analysis was conducted by removing the study by Rutkowski et al [41]. This study was excluded due to differences in disease severity, with the intervention and control groups having a significantly better baseline lung function compared with the other studies, potentially leading to a different treatment response. After excluding this study, heterogeneity reduced to 0%, and a fixed-effect model was applied. The results revealed a statistically significant improvement in FEV_1_ (%) between the intervention and comparator groups (MD 6.18, 95% CI 3.15-9.21, *P*<.001; Figure 7; see also [37,42,43]).

##### FEV1/FVC

**Figure 8:**
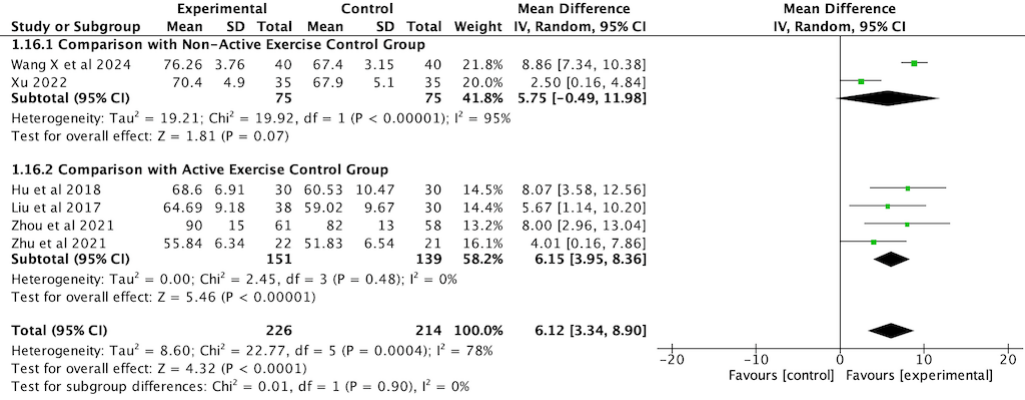
Forest plot of FEV1/FVC to assess lung function. FEV1: forced expiratory volume in 1 second; FVC: forced vital capacity.

Six RCTs [36,37,42,44,45,48] involving 440 participants used FEV_1_/FVC as an outcome measure. The subgroup analysis revealed a significant improvement in FEV_1_/FVC in the VR-complemented PR group compared with the active exercise controls (MD 6.15, 95% CI 3.95-8.36, *P*<.001) with low heterogeneity (*I*^2^=0%). By contrast, the improvement compared with nonactive exercise controls was not statistically significant (MD 5.75, 95% CI –0.49 to 11.98, *P*=.07), and there was high heterogeneity (*I*^2^=95%). The overall analysis revealed high heterogeneity across the studies (*I*^2^=78%, *P*<.001), likely due to variations in sample size, intervention duration, protocols, intensity, and types of interventions. Pooled results using a random-effects model indicated a significant improvement in FEV_1_/FVC in the intervention group compared with the comparator group (MD 6.12, 95% CI 3.34-8.90, *P*<.001; Figure 8; see also [36,37,42,44,45,48]).

**Figure 9:**
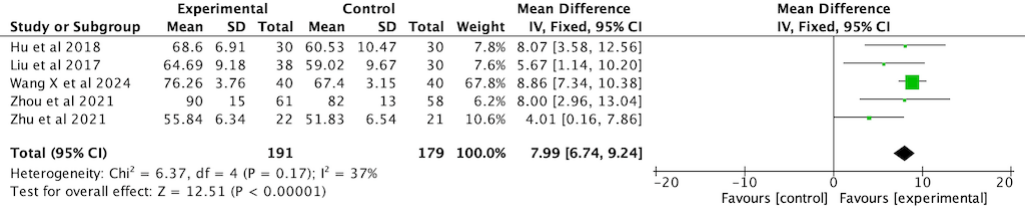
Forest plot of FEV1/FVC for lung function assessment after sensitivity analysis. FEV1: forced expiratory volume in 1 second; FVC: forced vital capacity.

Given the high heterogeneity, a sensitivity analysis was conducted. The study by Xu [45] was excluded due to differences in study design or data inconsistencies, which were likely contributing to the high heterogeneity. After removal of this study, heterogeneity decreased to a nonsignificant level (*I*^2^=37%, *P*=.17). Subsequently, a fixed-effects model was applied to the remaining studies. The revised analysis showed that the improvement in FEV_1_/FVC between the intervention and comparator groups remained significant (MD 7.99, 95% CI 6.74-9.24, *P*<.001; Figure 9; see also [36,37,42,44,48]).

##### FVC

**Figure 10:**
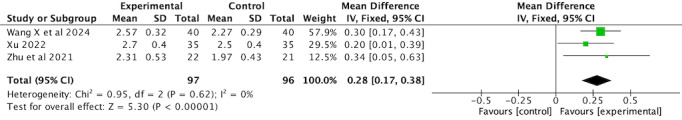
Forest plot of FVC to assess lung function. FVC: forced vital capacity.

Three RCTs [44,45,48] involving 193 participants evaluated FVC as an outcome measure. A low degree of heterogeneity was observed among these trials (*I*^2^=0%, *P*=.62). Pooled results using a fixed-effects model showed a significant difference in FVC in the intervention group compared with the comparator group (MD 0.28, 95% CI 0.17-0.38, *P*<.001; Figure 10; see also [44,45,48]).

#### Exercise Capacity

**Figure 11:**
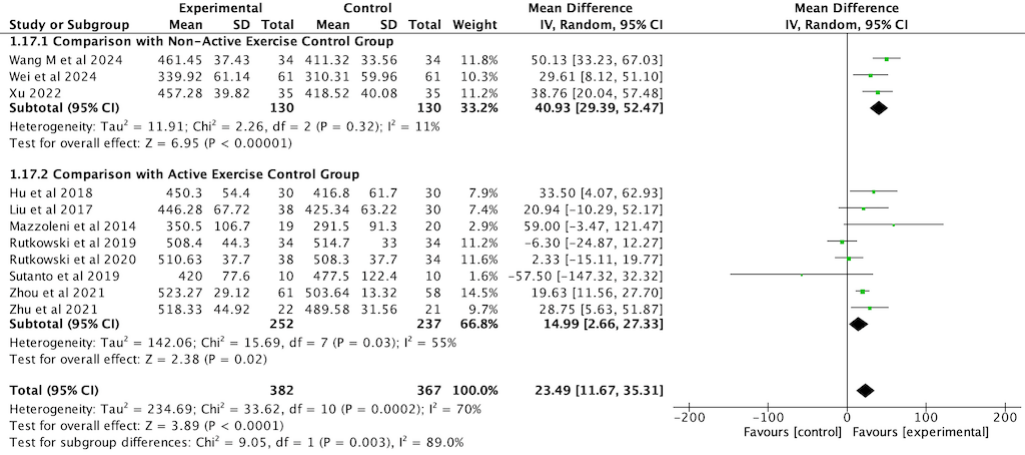
Forest plot of 6MWD to assess exercise capacity. 6MWD: 6-minute walk distance.

A total of 11 RCTs [35-40,42,44,45,49,50] involving 749 participants evaluated the 6MWD as an outcome measure. Significant improvements in 6MWD were observed in the VR-complemented PR group compared with both nonactive exercise controls and active exercise controls. The subgroup analysis revealed that the improvement in the VR group was more pronounced compared with nonactive exercise controls (MD 40.93, 95% CI 29.39-52.47, *P*<.001), with low heterogeneity (*I*^2^=11%). However, compared with active exercise controls, the effect size was smaller (MD 14.99, 95% CI 2.66-27.33, *P*=.02), with moderate heterogeneity (*I*^2^=55%). The overall analysis, pooling both subgroups, indicated a significant improvement in 6MWD for the intervention group over the control group (MD 23.49, 95% CI 11.67-35.31, *P*<.001). The random-effects model was applied due to the presence of moderate heterogeneity across studies (*I*^2^=70%; Figure 11; see also [35-40,42,44,45,49,50]), which reflects variations in study characteristics such as participant demographics, intervention protocols, and outcome measurement techniques.

**Figure 12:**
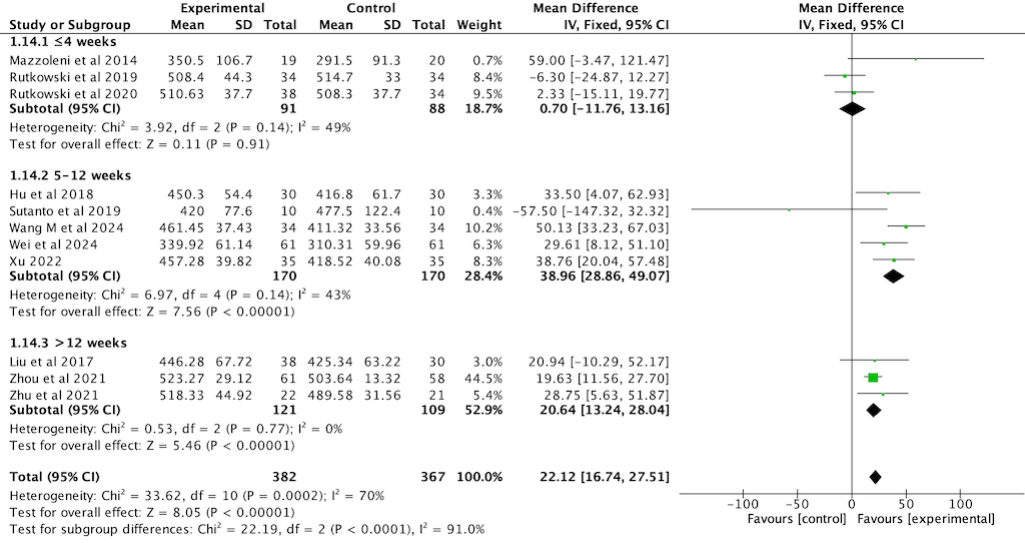
Forest plot of subgroup analysis of 6MWD by intervention duration. 6MWD: 6-minute walk distance.

A subgroup analysis based on the intervention duration was conducted to assess the impact of VR-complemented PR on 6MWD compared with the comparators. In the ≤4-week subgroup, no significant improvement in 6MWD was observed (MD 0.70, 95% CI −11.76 to 13.16, *P*=.91), with moderate heterogeneity (*I*^2^=49%). The 5-12-week subgroup demonstrated the largest effect size, with a significant improvement in 6MWD (MD 38.96, 95% CI 28.86-49.07, *P*<.001) and low heterogeneity (*I*^2^=43%). In the >12-week subgroup, a significant improvement was also noted (MD 20.64, 95% CI 13.24-28.04, *P*<.001), with no heterogeneity (*I*^2^=0%). A fixed-effects model was applied to all subgroups, considering the lower levels of heterogeneity observed in most categories (Figure 12; see also [35-40,42,44,45,49,50]).

A sensitivity analysis was conducted for 6MWD by sequentially removing each study to assess the robustness of the findings. The results remained consistent after the exclusion of any individual study, indicating that the overall effect was not driven by a single study.

#### Dyspnea

**Figure 13:**
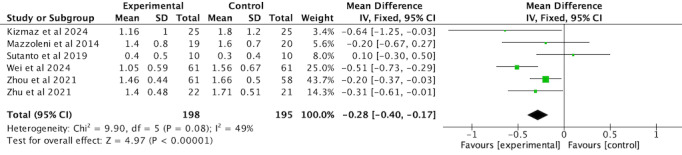
Forest plot of mMRC to assess dyspnea. mMRC: modified British Medical Research Council.

The mMRC scale, measured at rest, was used as an outcome measure in 6 RCTs [35,38,42,44,47,50] involving 393 participants. A moderate degree of heterogeneity was observed among the trials (*I*^2^=49%, *P*=.08); therefore, a fixed-effects model was applied. The pooled analysis showed a significant reduction in mMRC scores in the intervention group compared with the comparator group (MD –0.28, 95% CI –0.40 to –0.17, *P*<.001; Figure 13; see also [35,38,42,44,47,50]).

#### Health Status

**Figure 14:**
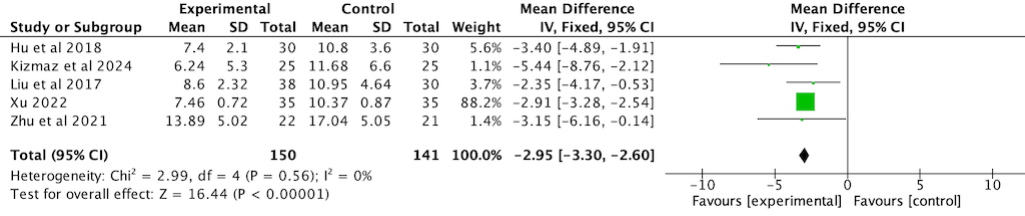
Forest plot of CAT to assess health status. CAT: COPD Assessment Test.

The effects of VR-complemented PR on the CAT score were reported in 5 RCTs [36,37,44,45,47] involving 291 patients with COPD. No significant heterogeneity was observed among the trials (*I*^2^=0%, *P*=.56); therefore, a fixed-effects model was applied. The analysis revealed that the intervention significantly improved the health status of patients with COPD compared with the comparator group (MD –2.95, 95% CI –3.30 to –2.60, *P*<.001; Figure 14; see also [36,37,44,45,47]).

#### Oxygenation Status

**Figure 15:**
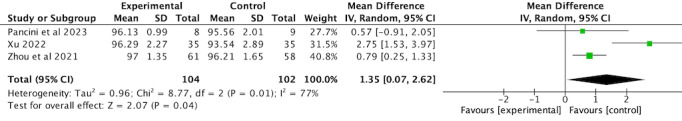
Forest plot of SpO2 to assess lung function. SpO2: peripheral capillary oxygen saturation.

Three RCTs [42,45,46] involving 206 participants evaluated SpO_2_, measured at rest, as an outcome measure. A high degree of heterogeneity was observed among the trials (*I*^2^=77%, *P*=.01), likely due to variations in sample size, intervention duration, protocols, intensity, and types of interventions. Pooled results using a random-effects model indicated that the intervention group showed a significant difference in SpO_2_ compared with the comparator group (MD 1.35, 95% CI 0.07-2.62, *P*=.04; Figure 15; see also [42,45,46]).

**Figure 16:**
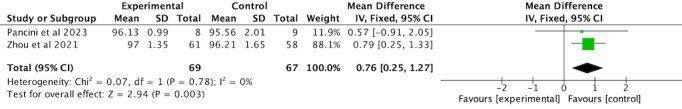
Forest plot of SpO2 for lung function assessment after sensitivity analysis. SpO2: peripheral capillary oxygen saturation.

Given the high heterogeneity, a sensitivity analysis was performed by excluding the study by Xu [45]. This study was excluded due to potential methodological differences or data inconsistencies that could have contributed to the observed heterogeneity. After excluding this study, heterogeneity was substantially reduced (*I*^2^=0%, *P*=.78). A fixed-effects model was applied, and the revised analysis showed that the significant difference in SpO_2_ between the intervention and comparator groups remained significant (MD 0.76, 95% CI 0.25-1.27, *P*=.003; Figure 16; see also [42,46]).

#### Patient-Reported Experience Measures

##### Acceptability

Two studies reported acceptability as an outcome measure. Mazzoleni et al [35] assessed acceptability using a 7-item questionnaire, where each item was scored on a 7-point Likert scale, with a total score range of 0-49. The results showed no significant difference in acceptability between the intervention and comparator groups (mean 42.4, SD 3.5 vs mean 43.9, SD 3.0, *P*=.17), indicating both protocols were well-tolerated. Xu [45] evaluated acceptability through a satisfaction questionnaire, categorizing responses as “satisfied” (≥90 points), “somewhat satisfied” (70-89 points), and “unsatisfied” (<70 points). Total satisfaction, defined as the proportion of “satisfied” and “somewhat satisfied” patients, was significantly higher in the intervention group compared with the comparator group (94.3% vs 77.1%, *P*=.04).

##### Engagement

Two studies reported engagement as an outcome measure. Zhou et al [42] assessed exercise adherence using the rate of achieving the exercise goal, defined as completing ≥5 sessions per week, each lasting ≥30 minutes, or accumulating ≥30 minutes per session through shorter bouts of ≥10 minutes. The intervention group had a significantly higher adherence rate than the comparator group (83.6% vs 67.2%, *P*<.05). Nonadherence reasons included lack of time, weather, forgetfulness, personal matters, travel, and in some cases, adverse effects such as knee pain or dyspnea after exercise. Similarly, Zhu et al [44] measured engagement using the exercise completion rate, defined as performing ≥4 sessions per week, each lasting ≥30 minutes. The intervention group again showed a significantly higher completion rate compared with the comparator group (90.9% vs 61.9%, *P*<.05). Reasons for noncompletion included travel and forgetfulness in the comparator group, while in the intervention group, joint pain and forgetfulness were noted.

## Discussion

### Principal Findings

This review included 16 eligible trials to summarize the evidence regarding the effects of VR-complemented PR compared with comparators in people with COPD. Compared with the previous meta-analysis by Chai et al [24], 5 additional studies were included, providing a more comprehensive and updated synthesis of evidence. The findings from this meta-analysis underscore the effectiveness of VR-complemented PR compared with comparators in improving a range of critical outcomes, including lung function (FEV_1_ [L], FEV_1_/FVC, FVC), exercise capacity (6MWD), dyspnea (mMRC), health status (CAT), oxygenation status (SpO_2_), and patient-reported experience measures (acceptability and engagement). Furthermore, subgroup analyses revealed that VR-complemented PR had a significantly greater effect on FEV_1_ (L) and 6MWD compared with nonactive exercise controls. Additionally, VR-complemented PR showed a greater improvement in FEV_1_/FVC compared with active exercise controls. The effectiveness of VR-complemented PR was also influenced by intervention duration, with the most significant improvements observed in programs lasting 5-12 weeks. However, sensitivity analyses indicated more consistent results after excluding certain studies. Despite this, the findings should be interpreted with caution due to the small sample sizes and limited number of trials, which may introduce bias and reduce generalizability.

### Lung Function

COPD is characterized by reduced lung function, which is strongly associated with an increased risk of exacerbations, hospitalization, and mortality [51,52]. Improving lung function is therefore a key goal of PR. In this meta-analysis, VR-complemented PR demonstrated significant improvements in FEV_1_ (L), FEV_1_/FVC, and FVC compared with comparator groups. Our results align with those reported by Chai et al [24], Liu et al [25], and Obrero-Gaitán et al [26], who similarly identified significant improvements in lung function following VR-based interventions compared with comparators. However, our meta-analysis expands upon these studies by incorporating a broader range of pulmonary outcomes and a larger data set, providing a more comprehensive synthesis of the evidence. Notably, the observed improvements in FEV_1_ (L) (MD 0.2 L) exceeded the widely accepted minimally clinically important difference (MCID) of 0.1 L [53], underscoring the clinical relevance of these changes. While specific MCID thresholds for FEV_1_/FVC and FVC are not well-established in the literature, the improvements observed in FEV_1_/FVC (MD 6.1%) and FVC (MD 0.3 L) suggest meaningful enhancements in lung function and capacity. By contrast, FEV_1_ (%) did not show a statistically significant improvement (MD 2.4%, *P*=.49), which may be attributed to the variability in baseline characteristics, treatment intensity, or measurement methods across studies [41]. Despite this, the improvements in other lung function measures (eg, FEV_1_ [L], FEV_1_/FVC, and FVC) suggest that VR-complemented PR may still have a beneficial effect on lung function, with further studies needed to better understand the specific impact on FEV_1_ (%).

The observed improvements in pulmonary function are likely driven by the enhanced physical activity, adherence, and engagement facilitated by the immersive and interactive features of VR technology [15]. By reducing the monotony of traditional PR and creating a more engaging and motivating environment, VR encourages consistent participation, leading to improved respiratory muscle strength and overall physical conditioning [54]. Furthermore, VR’s real-time feedback and personalized intensity adjustments enable patients to train within optimal zones, maximizing therapeutic benefits and contributing to the observed improvements in lung function.

### Subgroup Analysis of Lung Function

The subgroup analyses of FEV_1_ (L) and FEV_1_/FVC revealed contrasting results, emphasizing the complexity of evaluating lung function in patients with COPD. In the case of FEV_1_ (L), VR-complemented PR showed a significantly greater effect in the nonactive exercise control group (MD 0.3 L, *P*=.005) compared with the active exercise control group (MD 0.2 L, *P*=.05). This may suggest that VR-complemented PR could be more beneficial when combined with less intensive rehabilitation or usual care, possibly due to greater room for improvement in patients with lower baseline rehabilitation. However, further research is needed to confirm this potential benefit. These findings are consistent with Donath et al [55], who also found more pronounced VR effects in inactive control conditions. The clinical relevance of these results lies in the potential for VR to enhance rehabilitation in patients who are typically less engaged or receiving lower-intensity interventions, thus improving outcomes for a wider range of patients with COPD.

By contrast, the analysis of FEV_1_/FVC yielded different results. VR-complemented PR showed a significant improvement compared with the active exercise control group (MD 6.2%, *P*<.001), with no heterogeneity (*I*^2^=0%), indicating a robust effect. However, VR-complemented PR did not show a significant improvement compared with the nonactive exercise control group (MD 5.8%, *P*=.07), with high heterogeneity (*I*^2^=95%). These results may be due to the nature of FEV_1_/FVC as a ratio that is more sensitive to lung mechanics than absolute measures such as FEV_1_ (L) [56,57]. The lack of a significant result in the nonactive exercise control group may also reflect lower intervention intensity, limiting improvement.

### Exercise Capacity

Dysfunction and atrophy of skeletal muscle are common features of COPD, significantly impairing patients’ physical function and limiting their ability to perform daily activities. The 6MWD is a widely used measure of exercise capacity in COPD rehabilitation. In this meta-analysis, VR-complemented PR demonstrated a significant improvement in 6MWD compared with comparator groups, highlighting the effectiveness of VR in enhancing functional exercise capacity. Our findings align with those of Wang et al [22], Patsaki et al [23], Obrero-Gaitán et al [26], Chai et al [24], and Liu et al [25], who also reported significant improvements in 6MWD with VR-based interventions. The observed improvement in 6MWD (MD 23 m) approaches the MCID (mean 26 m, SD 2 m) for COPD [58], which suggests that while the improvement is statistically significant, it may not fully meet the threshold for clinical significance.

The subgroup analysis revealed that VR-complemented PR had a significantly greater effect (MD 41 m) on 6MWD compared with the nonactive exercise control group, a value that far exceeds the MCID [58]. This may indicate that VR interventions could be more beneficial when combined with less intensive rehabilitation or usual care, where there is more room for improvement. However, these findings are consistent with the results of Janhunen et al [59], but further research is needed to confirm this hypothesis. The engaging nature of VR likely enhances patient motivation and adherence, leading to more substantial gains in exercise capacity [60]. By contrast, VR-complemented PR showed a smaller effect size (MD 15 m) when compared with the active exercise control group, which is well below the MCID [58]. This suggests that the additional benefit of VR in settings with already structured exercise programs is more limited. The smaller effect may be due to the already high baseline exercise intensity in the active exercise control group, where the potential for further improvement is constrained.

A subgroup analysis based on intervention duration showed that VR-complemented PR is more effective with a 5-12-week duration compared with comparators. Programs of 5-12-week duration resulted in the largest improvements in 6MWD, while shorter interventions (≤4 weeks) showed no significant improvement. This is closer to the standard duration of PR (4-8 weeks) [29,30], suggesting that VR-complemented PR within this time frame may provide optimal improvements in exercise capacity. Shorter interventions (≤4 weeks) may not allow enough time for meaningful progress, while 5-12 weeks appears to be the most beneficial for improving physical function in patients with COPD.

The improvement may be attributed to VR’s interactive and engaging nature, which reduces the monotony of traditional PR, enhances adherence, and provides real-time feedback [61]. VR also creates an immersive environment that distracts patients from dyspnea and fatigue, enabling higher intensity and longer duration of exercise [62]. Moreover, VR’s personalized features allow tailored adjustments in exercise intensity and difficulty, making it adaptable to patients with varying physical capacities [63]. These factors collectively enhance skeletal muscle conditioning and overall physical function, directly supporting improved exercise capacity.

### Dyspnea

Dyspnea, a hallmark symptom of COPD, reflects an imbalance between ventilatory demand and capacity, significantly impacting patients’ quality of life and physical function [64]. In this meta-analysis, the mMRC scale, measured at rest, was used to evaluate dyspnea. The pooled analysis indicated a significant reduction in mMRC scores in the VR-complemented PR group compared with the comparator group, consistent with the findings of Chai et al [24]. However, our results differ from those of Patsaki et al [23], which included only 2 studies and did not show significant improvement. The broader inclusion criteria and larger data set in our analysis likely provide a more comprehensive understanding of VR-complemented PR’s effects on dyspnea.

The improvements in dyspnea can be attributed to VR’s immersive nature, which enhances exercise adherence by reducing boredom and distraction from breathlessness, enabling consistent training at higher intensities [65]. Physiologically, improved skeletal muscle function and exercise capacity lower ventilatory demand during activity, reducing dyspnea symptoms. Psychologically, VR’s engaging and gamified features alleviate anxiety and boost confidence, positively influencing breathlessness perception [66]. While the improvements in mMRC scores observed in our analysis are statistically significant, it is important to consider their clinical relevance. The modest reduction of 0.3 points, though notable, may have limited practical impact in isolation. However, when combined with significant gains in exercise capacity and health status, VR-complemented PR demonstrates a multifaceted approach to managing COPD symptoms, addressing both physical and psychological aspects of dyspnea.

### Health Status

Health status is a key outcome in COPD management, reflecting the disease’s impact on daily life and well-being. In this meta-analysis, VR-complemented PR significantly improved health status, as measured by CAT, with an MD of –3 compared with comparators. This reduction exceeds the MCID of 2 points, demonstrating both statistical and clinical relevance [67]. The included studies showed consistent results with no significant heterogeneity (*I*^2^=0%), reinforcing the reliability of these findings. Our results are in line with the review by Wang et al [22], which suggested that VR-complemented PR could improve CAT scores. However, the conclusions by Wang et al [22] were based on qualitative evidence, as they did not perform a meta-analysis and considered CAT as a measure of the quality of life. By contrast, our quantitative synthesis offers a more robust evaluation, strengthening the evidence base for the effectiveness of VR-complemented PR in improving health status.

The observed improvements can be attributed to the engaging and interactive nature of VR, which enhances adherence to PR programs by reducing monotony and providing real-time feedback. Better adherence leads to improved symptom management, reduced fatigue, and enhanced physical function [68]. Additionally, the immersive VR experience can alleviate anxiety and depression, common in patients with COPD, thereby contributing to a more positive perception of health [41]. Furthermore, VR’s adaptability allows for personalized rehabilitation tailored to individual needs and disease severity, ensuring that a broader range of patients can benefit. This comprehensive approach addresses both physical and psychological dimensions, underscoring the potential of VR-complemented PR as a valuable adjunct to conventional rehabilitation for improving health status in COPD.

### Oxygenation Status

SpO_2_, a measure of oxygen saturation in the blood, is an important indicator of respiratory function, especially in patients with COPD, who often experience oxygen desaturation during physical exertion [69]. In this meta-analysis, SpO_2_ was measured at rest, and our pooled analysis revealed a significant improvement in oxygenation status in the VR-complemented PR group compared with the comparator group (MD 1.35%). This finding suggests that VR-complemented PR has the potential to enhance oxygenation in patients with COPD, contributing to overall respiratory health. Similarly, Condon et al [70] explored the effectiveness of VR gaming and exercise-based games for patients with respiratory disease and reported a significant increase in SpO_2_ compared with the control group (standardized MD 0.2%).

The immersive nature of VR likely enhances engagement and motivates patients to perform more sustained and intense physical activity, which can help improve respiratory efficiency [71]. By encouraging higher-intensity exercise, VR facilitates greater use of lung capacity, which in turn may reduce the frequency and severity of desaturation events during physical exertion [72]. Additionally, VR interventions provide real-time biofeedback, allowing patients to monitor their respiratory patterns and adjust breathing techniques. This feedback helps them maintain controlled, efficient breathing during exercise, reducing the risk of rapid, shallow breathing, a common cause of desaturation in patients with COPD [73]. Moreover, VR’s interactive environment provides a more engaging and motivating rehabilitation experience compared with traditional methods, which may lead to more consistent participation and, consequently, better exercise outcomes [74]. By enhancing patient motivation and adherence, VR can foster improvements in both physical capacity and respiratory efficiency, ultimately contributing to more stable oxygen saturation levels during activity [75].

### Acceptability and Engagement

Acceptability and engagement are crucial for the success of PR programs. In this review, VR-complemented PR demonstrated good acceptability, with most patients reporting positive experiences. While 1 study found no significant difference compared with traditional PR, another reported higher satisfaction in the VR group. The immersive and interactive nature of VR likely reduces boredom and increases motivation, making rehabilitation more appealing.

Engagement, assessed through adherence rates, was consistently higher in VR-complemented PR groups. Features such as real-time feedback, gamification, and personalized difficulty levels help sustain interest and participation [61]. These findings are consistent with previous systematic reviews, which also reported that participants enjoyed VR technology and demonstrated good adherence [22,23]. VR addresses common barriers to adherence, such as monotony and lack of motivation, by transforming exercise into an engaging experience. However, challenges such as joint pain and forgetfulness still impact adherence and should be addressed through tailored strategies [44].

### Strengths, Limitations, and Future Research Directions

This review has several notable strengths. First, it is one of the most comprehensive systematic reviews and meta-analyses on VR-complemented PR for COPD, incorporating a rigorous search strategy across multiple international and Chinese databases. The inclusion of recent studies offers a robust and up-to-date synthesis of evidence. Second, stringent inclusion and exclusion criteria were applied to ensure that only high-quality RCTs were analyzed, enhancing the reliability of the findings. Third, the use of subgroup analyses, which examined factors such as comparator type and intervention duration, provided a nuanced understanding of the conditions under which VR interventions are most effective, offering valuable insights for clinical practice. Additionally, sensitivity analyses were conducted to address heterogeneity, reinforcing the robustness and consistency of the results. Finally, the assessment of patient-reported experience measures, such as acceptability and engagement, offers a holistic perspective on the intervention’s feasibility and its impact on patient-centered outcomes.

Several limitations should be acknowledged. Blinding of participants and personnel was not feasible due to the nature of VR interventions, increasing the risk of performance bias. The inclusion of studies with PR durations shorter than standard recommendations [29,30] may limit comparability to conventional PR programs. While sensitivity analyses excluding these shorter-duration studies confirmed the robustness of the findings, this limitation highlights the need for future research adhering to standardized PR protocols. Additionally, the focus on hospital-based VR programs may reduce the generalizability of the findings to home-based settings, where factors such as environmental differences and the availability of supervision could influence outcomes. A potential limitation is the possible overlap of participant samples in Rutkowski et al [39] and Rutkowski et al [40], as both studies reported identical mean age and disease severity for their comparator groups. While the intervention groups and objectives differed, this overlap may have introduced bias. Sensitivity analyses excluding 1 study confirmed the robustness of the findings, but the possibility of partial overlap should be considered when interpreting the results. Finally, while the subgroup analyses provided valuable insights into the effectiveness of VR-complemented PR, their findings should be interpreted with caution. The subgroup sizes were relatively small, limiting statistical power and increasing the potential for type I or type II errors.

Future research should aim to address these limitations by adopting more rigorous and standardized study designs. Specifically, studies with larger sample sizes and longer follow-up periods are needed to evaluate the long-term effectiveness and sustainability of VR-complemented PR. Developing standardized VR protocols that define exercise intensity, frequency, duration, and content would help reduce heterogeneity and improve the comparability of results across studies. Expanding research to include home-based VR programs is also essential, as this could enhance accessibility, reduce the burden of travel, and improve adherence, particularly for patients with mobility issues. Additionally, future studies should explore the effects of VR-complemented PR on diverse patient subgroups to identify those who may benefit the most from this intervention. Comprehensive evaluation indicators, such as psychological function, cognitive function, and frailty status, should be included to assess the full range of potential benefits.

### Conclusions

This systematic review and meta-analysis demonstrate that VR-complemented PR effectively improves lung function, exercise capacity, dyspnea, health status, and oxygenation status compared with comparators in patients with COPD. The engaging and immersive nature of VR enhances patient adherence and participation, addressing key limitations of traditional PR. Subgroup analyses revealed that VR-complemented PR had a significantly greater effect on FEV_1_ (L) and 6MWD when compared with the nonactive exercise control groups. Additionally, VR-complemented PR showed a greater improvement in FEV_1_/FVC compared with the active exercise control groups. The effectiveness of VR interventions in 6MWD also varied with intervention duration, with the most pronounced benefits observed in programs lasting 5-12 weeks. The findings underscore the potential of VR as an innovative, patient-centered adjunct to traditional PR. Future studies should focus on long-term outcomes, standardized protocols, and the applicability of VR in diverse and home-based settings to optimize its clinical implementation.

## Appendix

Multimedia Appendix 1 Search strategy.

Multimedia Appendix 2 PRISMA checklist.

## Abbreviations

6MWD: 6-minute walk distance
CAT: COPD Assessment Test
COPD: chronic obstructive pulmonary disease
FEV1: forced expiratory volume in 1 second
FVC: forced vital capacity
GOLD: Global Initiative for Chronic Obstructive Lung Disease
HRQoL: health-related quality of life
MCID: minimally clinically important difference
MD: mean difference
MeSH: Medical Subject Headings
mMRC: modified British Medical Research Council
PICOS: Participant, Intervention, Comparator, Outcomes, and Study Design
PR: pulmonary rehabilitation
PRISMA: Preferred Reporting Items for Systematic Reviews and Meta-Analyses
RCT: randomized controlled trial
SpO2: peripheral capillary oxygen saturation
VR: virtual reality

## Data Availability

All the relevant data used and generated during this study are included in the manuscript.

## References

[1] Halpin DMG, Tashkin DP (2009). Defining disease modification in chronic obstructive pulmonary disease. COPD.

[2] Jácome Cristina, Marques A (2014). Pulmonary rehabilitation for mild COPD: a systematic review. Respir Care.

[3] Rochester CL, Alison JA, Carlin B, Jenkins AR, Cox NS, Bauldoff G, Bhatt SP, Bourbeau J, Burtin C, Camp PG, Cascino TM, Dorney Koppel GA, Garvey C, Goldstein R, Harris D, Houchen-Wolloff L, Limberg T, Lindenauer PK, Moy ML, Ryerson CJ, Singh SJ, Steiner M, Tappan RS, Yohannes AM, Holland AE (2023). Pulmonary rehabilitation for adults with chronic respiratory disease: an official American Thoracic Society clinical practice guideline. Am J Respir Crit Care Med.

[4] He W, Wang J, Feng Z, Li J, Xie Y (2023). Effects of exercise-based pulmonary rehabilitation on severe/very severe COPD: a systematic review and meta-analysis. Ther Adv Respir Dis.

[5] Higashimoto Yuji, Ando Morihide, Sano Akiko, Saeki Sho, Nishikawa Yusaku, Fukuda Kanji, Tohda Yuji (2020). Effect of pulmonary rehabilitation programs including lower limb endurance training on dyspnea in stable COPD: a systematic review and meta-analysis. Respir Investig.

[6] Bhatt SP, Westra J, Kuo Y, Sharma G (2024). Pulmonary rehabilitation utilization in older adults with chronic obstructive pulmonary disease, 2013-2019. Ann Am Thorac Soc.

[7] Spitzer KA, Stefan MS, Priya A, Pack QR, Pekow PS, Lagu T, Pinto-Plata VM, ZuWallack RL, Lindenauer PK (2019). Participation in pulmonary rehabilitation after hospitalization for chronic obstructive pulmonary disease among medicare beneficiaries. Ann Am Thorac Soc.

[8] Jones SE, Green SA, Clark AL, Dickson MJ, Nolan A, Moloney C, Kon SSC, Kamal F, Godden J, Howe C, Bell D, Fleming S, Haselden BM, Man WD (2014). Pulmonary rehabilitation following hospitalisation for acute exacerbation of COPD: referrals, uptake and adherence. Thorax.

[9] Almadana Pacheco V, Pavón Masa María, Gómez-Bastero Fernández Ana Paulina, Muñiz Rodríguez Ana Mirian, Tallón Moreno Rodrigo, Montemayor Rubio T (2017). Patient profile of drop-outs from a pulmonary rehabilitation program. Arch Bronconeumol.

[10] Hayton C, Clark A, Olive S, Browne P, Galey P, Knights E, Staunton L, Jones A, Coombes E, Wilson AM (2013). Barriers to pulmonary rehabilitation: characteristics that predict patient attendance and adherence. Respir Med.

[11] Pierobon A, Sini Bottelli E, Ranzini L, Bruschi C, Maestri R, Bertolotti G, Sommaruga M, Torlaschi V, Callegari S, Giardini A (2017). COPD patients' self-reported adherence, psychosocial factors and mild cognitive impairment in pulmonary rehabilitation. Int J Chron Obstruct Pulmon Dis.

[12] McNamara RJ, Dale M, McKeough ZJ (2019). Innovative strategies to improve the reach and engagement in pulmonary rehabilitation. J Thorac Dis.

[13] Birckhead B, Khalil C, Liu X, Conovitz S, Rizzo A, Danovitch I, Bullock K, Spiegel B (2019). Recommendations for methodology of virtual reality clinical trials in health care by an international working group: iterative study. JMIR Ment Health.

[14] Rutkowski S (2021). Management challenges in chronic obstructive pulmonary disease in the COVID-19 pandemic: telehealth and virtual reality. J Clin Med.

[15] Shi L, Liu F, Liu Y, Wang R, Zhang J, Zhao Z, Zhao J (2023). Biofeedback respiratory rehabilitation training system based on virtual reality technology. Sensors (Basel).

[16] Pourmand A, Davis S, Lee D, Barber S, Sikka N (2017). Emerging utility of virtual reality as a multidisciplinary tool in clinical medicine. Games Health J.

[17] Chen J, Or CK, Chen T (2022). Effectiveness of using virtual reality-supported exercise therapy for upper extremity motor rehabilitation in patients with stroke: systematic review and meta-analysis of randomized controlled trials. J Med Internet Res.

[18] Wu Y, Wang N, Zhang H, Sun X, Wang Y, Zhang Y (2023). Effectiveness of virtual reality in symptom management of cancer patients: a systematic review and meta-analysis. J Pain Symptom Manage.

[19] Liu C, Wang X, Chen R, Zhang J (2022). The effects of virtual reality training on balance, gross motor function, and daily living ability in children with cerebral palsy: systematic review and meta-analysis. JMIR Serious Games.

[20] Lei C, Sunzi K, Dai F, Liu X, Wang Y, Zhang B, He L, Ju M (2019). Effects of virtual reality rehabilitation training on gait and balance in patients with Parkinson's disease: a systematic review. PLoS One.

[21] De Miguel-Rubio A, Rubio MD, Alba-Rueda A, Salazar A, Moral-Munoz JA, Lucena-Anton D (2020). Virtual reality systems for upper limb motor function recovery in patients with spinal cord injury: systematic review and meta-analysis. JMIR Mhealth Uhealth.

[22] Wang YQ, Liu X, Ma RC, Yin YY, Yang Z, Cao HP, Xie J (2020). Active video games as an adjunct to pulmonary rehabilitation of patients with chronic obstructive pulmonary disease: a systematic review and meta-analysis. Am J Phys Med Rehabil.

[23] Patsaki I, Avgeri V, Rigoulia T, Zekis T, Koumantakis GA, Grammatopoulou E (2023). Benefits from incorporating virtual reality in pulmonary rehabilitation of COPD patients: a systematic review and meta-analysis. Adv Respir Med.

[24] Chai X, Wu L, He Z (2023). Effects of virtual reality-based pulmonary rehabilitation in patients with chronic obstructive pulmonary disease: a meta-analysis. Medicine (Baltimore).

[25] Liu Y, Du Q, Jiang Y (2024). The effect of virtual reality technology in exercise and lung function of patients with chronic obstructive pulmonary disease: a systematic review and meta-analysis. Worldviews Evid Based Nurs.

[26] Obrero-Gaitán Esteban, Chau-Cubero CY, Lomas-Vega R, Osuna-Pérez María Catalina, García-López Héctor, Cortés-Pérez Irene (2024). Effectiveness of virtual reality-based therapy in pulmonary rehabilitation of chronic obstructive pulmonary disease. A systematic review with meta-analysis. Heart Lung.

[27] Xie X, Fan J, Chen H, Zhu L, Wan T, Zhou J, Fan D, Hu X (2021). Virtual reality technology combined with comprehensive pulmonary rehabilitation on patients with stable chronic obstructive pulmonary disease. J Healthc Eng.

[28] Hutton B, Catalá-López Ferrán, Moher D (2016). [The PRISMA statement extension for systematic reviews incorporating network meta-analysis: PRISMA-NMA]. Med Clin (Barc).

[29] Spruit MA, Singh SJ, Garvey C, ZuWallack R, Nici L, Rochester C, Hill K, Holland AE, Lareau SC, Man WD, Pitta F, Sewell L, Raskin J, Bourbeau J, Crouch R, Franssen FME, Casaburi R, Vercoulen JH, Vogiatzis I, Gosselink R, Clini EM, Effing TW, Maltais F, van der Palen Job, Troosters T, Janssen DJA, Collins E, Garcia-Aymerich J, Brooks D, Fahy BF, Puhan MA, Hoogendoorn M, Garrod R, Schols AMWJ, Carlin B, Benzo R, Meek P, Morgan M, Rutten-van Mölken Maureen P M H, Ries AL, Make B, Goldstein RS, Dowson CA, Brozek JL, Donner CF, Wouters EFM, ATS/ERS Task Force on Pulmonary Rehabilitation (2013). An official American Thoracic Society/European Respiratory Society statement: key concepts and advances in pulmonary rehabilitation. Am J Respir Crit Care Med.

[30] McCarthy B, Casey D, Devane D, Murphy K, Murphy E, Lacasse Y (2015). Pulmonary rehabilitation for chronic obstructive pulmonary disease. Cochrane Database Syst Rev.

[31] (GOLD) G Global strategy for prevention, diagnosis and management of COPD: 2025 report. The Global Initiative for Chronic Obstructive Lung Disease.

[32] McGrath S, Zhao X, Steele R, Thombs BD, Benedetti A, DEPRESsion Screening Data (DEPRESSD) Collaboration (2020). Estimating the sample mean and standard deviation from commonly reported quantiles in meta-analysis. Stat Methods Med Res.

[33] Chi KY, Li MY, Chen C, Kang E, Cochrane Taiwan (2023). Ten circumstances and solutions for finding the sample mean and standard deviation for meta-analysis. Syst Rev.

[34] Higgins JPT, Altman DG, Gøtzsche Peter C, Jüni Peter, Moher D, Oxman AD, Savovic J, Schulz KF, Weeks L, Sterne JAC, Cochrane Bias Methods Group, Cochrane Statistical Methods Group (2011). The Cochrane Collaboration's tool for assessing risk of bias in randomised trials. BMJ.

[35] Mazzoleni Stefano, Montagnani Giulia, Vagheggini Guido, Buono Lorenzo, Moretti Francesca, Dario Paolo, Ambrosino Nicolino (2014). Interactive videogame as rehabilitation tool of patients with chronic respiratory diseases: preliminary results of a feasibility study. Respir Med.

[36] Liu P, Liu Q, Han W, Sun SP (2017). Effect of virtual reality technology combined with comprehensive pulmonary rehabilitation on stable stage COPD in the patients. Int J Geriatr.

[37] Hu DD, He J, Ding YQ, Xu J, Zhu HY (2018). Application effect of virtual reality technology in the pulmonary rehabilitation program for elderly COPD patients complicated with mild cognitive impairment. Pract J Cardiac Cereb Pneum Vasc Dis.

[38] Sutanto YS, Makhabah DN, Aphridasari J, Doewes M, Ambrosino N, Suradi (2019). Videogame assisted exercise training in patients with chronic obstructive pulmonary disease: a preliminary study. Pulmonology.

[39] Rutkowski S, Rutkowska A, Jastrzębski D, Racheniuk H, Pawełczyk W, Szczegielniak J (2019). Effect of virtual reality-based rehabilitation on physical fitness in patients with chronic obstructive pulmonary disease. J Hum Kinet.

[40] Rutkowski S, Rutkowska A, Kiper P, Jastrzebski D, Racheniuk H, Turolla A, Szczegielniak J, Casaburi R (2020). Virtual reality rehabilitation in patients with chronic obstructive pulmonary disease: a randomized controlled trial. COPD.

[41] Rutkowski S, Szczegielniak J, Szczepańska-Gieracha J (2021). Evaluation of the efficacy of immersive virtual reality therapy as a method supporting pulmonary rehabilitation: a randomized controlled trial. J Clin Med.

[42] Zhou TM, Zhang YL, Zhu YY (2021). Effects of exercise program based on somatosensory interactive games in elderly patients with stable COPD. Chin J Mod Nurs.

[43] Liu HL, Yang XN, Wang XK, Yang XS, Zhang XS, Li Q (2021). Study on adjuvant medication for patients with mild cognitive impairment based on VR technology and health education. Contrast Media Mol Imaging.

[44] Zhu Y, Xie H, Zhang L (2021). Clinical effect of applying somatosensory games based on Kinect 2.0 system to elderly patients with chronic obstructive pulmonary disease. J Wannan Medical College.

[45] Xu CJ (2022). Effect of virtual scene rehabilitation technology combined with comprehensive respiratory training in elderly patients with COPD. Chinese and Foreign Medical Research.

[46] Pancini E, Fumagalli A, Maggiolini S, Misuraca C, Negri D (2023). Promoting emotional and psychological well-being of patients with chronic obstructive pulmonary disease: A feasibility study combining virtual reality and savoring. Ann Rev CyberTherapy Telemed.

[47] Kizmaz E, Telli Atalay O, Çetin Nazlı, Uğurlu E (2024). Virtual reality for COPD exacerbation: a randomized controlled trial. Respir Med.

[48] Wang X, Zheng ZP, Qiu SS (2024). Impacts of biomaster virtual scenario interactive rehabilitation combined with multiple respiratory rehabilitation training on cardiopulmonary function and respiratory function in elderly patients with chronic obstructive pulmonary disease. Chin J Convalescent Med.

[49] Wang MF, Wan HM, Wang YQ (2024). The impact of vr technology-assisted cognitive behavioral nursing on fear of dyspnea in COPD patients. Guizhou Medical Journal.

[50] Wei HM, Wang JH, Wang XY (2024). The effects of somatosensory interactive games on dyspnea, balance function and exercise ability in patients with acute exacerbation of COPD were observed. Dec 20, 2024.

[51] Martin AL, Marvel J, Fahrbach K, Cadarette SM, Wilcox TK, Donohue JF (2016). The association of lung function and St. George's respiratory questionnaire with exacerbations in COPD: a systematic literature review and regression analysis. Respir Res.

[52] Garcia-Aymerich J, Serra Pons I, Mannino DM, Maas AK, Miller DP, Davis KJ (2011). Lung function impairment, COPD hospitalisations and subsequent mortality. Thorax.

[53] Jones PW, Beeh KM, Chapman KR, Decramer M, Mahler DA, Wedzicha JA (2014). Minimal clinically important differences in pharmacological trials. Am J Respir Crit Care Med.

[54] Wardini R, Dajczman E, Yang N, Baltzan M, Préfontaine David, Stathatos M, Marciano H, Watson S, Wolkove N (2013). Using a virtual game system to innovate pulmonary rehabilitation: safety, adherence and enjoyment in severe chronic obstructive pulmonary disease. Can Respir J.

[55] Donath L, Rössler Roland, Faude O (2016). Effects of virtual reality training (exergaming) compared to alternative exercise training and passive control on standing balance and functional mobility in healthy community-dwelling seniors: a meta-analytical review. Sports Med.

[56] Langan RC, Goodbred AJ (2020). Office spirometry: indications and interpretation. Am Fam Physician.

[57] Culver BH (2011). Obstructive? Restrictive? Or a ventilatory impairment?. Chest.

[58] Puhan MA, Chandra D, Mosenifar Z, Ries A, Make B, Hansel NN, Wise RA, Sciurba F, National Emphysema Treatment Trial (NETT) Research Group (2011). The minimal important difference of exercise tests in severe COPD. Eur Respir J.

[59] Janhunen M, Karner V, Katajapuu N, Niiranen O, Immonen J, Karvanen J, Heinonen A, Aartolahti E (2021). Effectiveness of exergame intervention on walking in older adults: a systematic review and meta-analysis of randomized controlled trials. Phys Ther.

[60] Aderinto N, Olatunji G, Abdulbasit MO, Edun M, Aboderin G, Egbunu E (2023). Exploring the efficacy of virtual reality-based rehabilitation in stroke: a narrative review of current evidence. Ann Med.

[61] Lan K, Li C, Cheung Y (2021). Slow breathing exercise with multimodal virtual reality: a feasibility study. Sensors (Basel).

[62] Colombo V, Mondellini M, Fumagalli A, Aliverti A, Sacco M (2024). A virtual reality-based endurance training program for COPD patients: acceptability and user experience. Disabil Rehabil Assist Technol.

[63] McAnirlin O, Browning M, Fasolino T, Okamoto K, Sharaievska I, Thrift J, Pope J (2024). Co-creating and delivering personalized, nature-based VR experiences: proof-of-concept study with four U.S. adults living with severe COPD. Wellbeing, Space and Society.

[64] James MD, Phillips DB, Vincent SG, Abdallah SJ, Donovan AA, de-Torres JP, Neder JA, Smith BM, Jensen D, O'Donnell DE, Canadian Respiratory Research Network (2022). Exertional dyspnoea in patients with mild-to-severe chronic obstructive pulmonary disease: neuromechanical mechanisms. J Physiol.

[65] Nicolas S, Beaumont M, Glaziou P, Simon B, Gut-Gobert C, Couturaud F (2021). Comparison of the cardio-respiratory response of a training session on cycloergometer or treadmill versus an Adapted Physical Activity session on Nintendo Wii, in patients with chronic respiratory disease. Respir Med Res.

[66] Jung T, Moorhouse N, Shi X, Amin MF (2020). A virtual reality-supported intervention for pulmonary rehabilitation of patients with chronic obstructive pulmonary disease: mixed methods study. J Med Internet Res.

[67] Smid DE, Franssen FME, Houben-Wilke S, Vanfleteren LEGW, Janssen DJA, Wouters EFM, Spruit MA (2017). Responsiveness and MCID estimates for CAT, CCQ, and HADS in patients with COPD undergoing pulmonary rehabilitation: a prospective analysis. J Am Med Dir Assoc.

[68] Gabriel AS, Tsai TY, Xhakli T, Finkelstein J (2023). Patient perceptions of a virtual reality-based system for pulmonary rehabilitation: a qualitative analysis. Stud Health Technol Inform.

[69] García-Talavera Ignacio, Jiménez González Patricia, Dorta Sánchez Rafael (2015). Exercise-induced oxygen desaturation in chronic obstructive pulmonary disease patients. Arch Bronconeumol.

[70] Condon C, Lam WT, Mosley C, Gough S (2020). A systematic review and meta-analysis of the effectiveness of virtual reality as an exercise intervention for individuals with a respiratory condition. Adv Simul (Lond).

[71] Barbour B, Sefton L, Bruce RM, Valmaggia L, Runswick OR (2024). Acute psychological and physiological benefits of exercising with virtual reality. PLoS One.

[72] Nielsen HB (2003). Arterial desaturation during exercise in man: implication for O2 uptake and work capacity. Scand J Med Sci Sports.

[73] Alhammad SA (2024). Advocating for action: exploring the potential of virtual reality in breathing exercise - a review of the clinical applications. Patient Prefer Adherence.

[74] Naqvi WM, Naqvi I, Mishra GV, Vardhan V (2024). The dual importance of virtual reality usability in rehabilitation: a focus on therapists and patients. Cureus.

[75] Blum J, Rockstroh C, Göritz Anja S (2020). Development and pilot test of a virtual reality respiratory biofeedback approach. Appl Psychophysiol Biofeedback.

