# Supplementary material for: Effectiveness of Virtual Reality–Complemented Pulmonary Rehabilitation on Lung Function, Exercise Capacity, Dyspnea, and Health Status in Chronic Obstructive Pulmonary Disease: Systematic Review and Meta-Analysis
Source: J Med Internet Res. 2025 Apr 7;27:e64742. doi: 10.2196/64742 (PMC12012404; doi:10.2196/64742)
Supplement: Multimedia Appendix 1 [file jmir_v27i1e64742_app1.docx]

# Appendix 1. Search strategy

This document outlines the detailed search strategy employed to identify relevant studies from the earliest date available until November 2024

## PubMed

| No. | Searches | Results |
| --- | --- | --- |
| #1 | (((((((((((((((((virtual reality[MeSH Terms]) OR (VR[Title/Abstract])) OR (virtual environment[Title/Abstract])) OR (video game*[Title/Abstract])) OR (virtual simulation[Title/Abstract])) OR (virtual medicine[Title/Abstract])) OR (mixed reality[Title/Abstract])) OR (commercial game*[Title/Abstract])) OR (virtual game*[Title/Abstract])) OR (exergam*[Title/Abstract])) OR (play-based therapy[Title/Abstract])) OR (augmented reality[Title/Abstract])) OR (virtual reality exposure therapy[Title/Abstract])) OR (x-box 360[Title/Abstract])) OR (kinect[Title/Abstract])) OR (wii[Title/Abstract])) OR (virtual world[Title/Abstract])) OR (head-mounted display[Title/Abstract]) | 37,662 |
| #2 | ((((((((pulmonary disease, chronic obstructive[MeSH Terms]) OR (chronic obstructive pulmonary disease*[Title/Abstract])) OR (chronic obstructive airway disease[Title/Abstract])) OR (chronic obstructive lung disease[Title/Abstract])) OR (COAD[Title/Abstract])) OR (COPD[Title/Abstract])) OR (airflow obstruction, chronic[Title/Abstract])) OR (airflow obstructions, chronic[Title/Abstract])) OR (chronic airflow obstruction*[Title/Abstract]) | 113,625 |
| #3 | #1 AND #2 | 89 |

## Web of Science

| No. | Searches | Results |
| --- | --- | --- |
| #1 | TS=(virtual reality) OR TS=(VR) OR TS=(virtual environment) OR TS=(video game*) OR TS=(virtual simulation) OR TS=(virtual medicine) OR TS=(mixed reality) OR TS=(commercial game*) OR TS=(virtual game*) OR TS=(exergam*) OR TS=(play-based therapy) OR TS=(augmented reality) OR TS=(virtual reality exposure therapy) OR TS=(x-box 360) OR TS=(kinect) OR TS=(wii) OR TS=(virtual world) OR TS=(head-mounted display) | 319,302 |
| #2 | TS=(pulmonary disease, chronic obstructive) OR TS=(chronic obstructive pulmonary disease* ) OR TS=(chronic obstructive airway disease) OR TS=(chronic obstructive lung disease ) OR TS=(COAD) OR TS=(COPD) OR TS=( chronic airflow obstruction* ) OR TS=( airflow obstruction, chronic) OR TS=(airflow obstructions, chronic ) | 120,707 |
| #3 | #1 AND #2 | 183 |

## CINAHL

| No. | Searches | Results |
| --- | --- | --- |
| #1 | SU virtual reality OR SU VR OR SU virtual environment OR SU video game* OR SU virtual simulation OR SU virtual medicine OR SU mixed reality OR SU commercial game* OR SU virtual game* OR SU exergam* OR SU play-based therapy OR SU augmented reality OR SU virtual reality exposure therapy OR SU x-box 360 OR SU kinect OR SU wii OR SU virtual world OR SU head-mounted display | 25,504 |
| #2 | SU pulmonary disease, chronic obstructive OR SU chronic obstructive pulmonary disease* OR SU chronic obstructive airway disease OR SU chronic obstructive lung disease OR SU COAD OR SU COPD OR SU chronic airflow obstruction* OR SU airflow obstruction,chronic OR SU airflow obstructions, chronic | 44,948 |
| #3 | #1 AND #2 | 28 |

## PsycINFO

| No. | Searches | Results |
| --- | --- | --- |
| #1 | SU virtual reality OR SU VR OR SU virtual environment OR SU video game* OR SU virtual simulation OR SU virtual medicine OR SU mixed reality OR SU commercial game* OR SU virtual game* OR SU exergam* OR SU play-based therapy OR SU augmented reality OR SU virtual reality exposure therapy OR SU x-box 360 OR SU kinect OR SU wii OR SU virtual world OR SU head-mounted display | 86,741 |
| #2 | SU pulmonary disease, chronic obstructive OR SU chronic obstructive pulmonary disease* OR SU chronic obstructive airway disease OR SU chronic obstructive lung disease OR SU COAD OR SU COPD OR SU chronic airflow obstruction* OR SU airflow obstruction, chronic OR SU airflow obstructions, chronic | 8,514 |
| #3 | #1 AND #2 | 9 |

## EMBASE

| No. | Searches | Results |
| --- | --- | --- |
| #1 | 'virtual reality'/exp OR vr:ti,ab,kw OR 'virtual environment':ti,ab,kw OR 'video game*':ti,ab,kw OR 'virtual simulation':ti,ab,kw OR 'virtual medicine':ti,ab,kw OR 'mixed reality':ti,ab,kw OR 'commercial game*':ti,ab,kw OR 'virtual game*':ti,ab,kw OR exergam*:ti,ab,kw OR 'play-based therapy':ti,ab,kw OR 'augmented reality':ti,ab,kw OR 'virtual reality exposure therapy':ti,ab,kw OR 'x-box 360':ti,ab,kw OR kinect:ti,ab,kw OR wii:ti,ab,kw OR 'virtual world':ti,ab,kw OR 'head-mounted display':ti,ab,kw | 62,296 |
| #2 | 'pulmonary disease, chronic obstructive'/exp OR 'chronic obstructive pulmonary disease*':ti,ab,kw OR 'chronic obstructive airway disease':ti,ab,kw OR 'chronic obstructive lung disease':ti,ab,kw OR coad:ti,ab,kw OR copd:ti,ab,kw OR 'chronic airflow obstruction*':ti,ab,kw OR 'airflow obstruction, chronic':ti,ab,kw OR 'airflow obstructions, chronic':ti,ab,kw | 224,376 |
| #3 | #1 AND #2 | 214 |

## The Cochrane Library

| No. | Searches | Results |
| --- | --- | --- |
| #1 | (virtual reality):ti,ab,kw OR (VR):ti,ab,kw OR (virtual environment):ti,ab,kw OR (video game*):ti,ab,kw OR (commercial game*):ti,ab,kw OR (virtual simulation):ti,ab,kw OR (virtual medicine):ti,ab,kw OR (mixed reality):ti,ab,kw | 12,920 |
| #2 | (virtual game*):ti,ab,kw OR (exergam*):ti,ab,kw OR (play-based therapy):ti,ab,kw OR (augmented reality):ti,ab,kw OR (virtual reality exposure therapy):ti,ab,kw | 3,694 |
| #3 | (x-box 360):ti,ab,kw OR (kinect):ti,ab,kw OR (wii):ti,ab,kw OR (virtual world):ti,ab,kw OR (head-mounted display):ti,ab,kw | 2,723 |
| #4 | #1 OR #2 OR #3 | 14,872 |
| #5 | (pulmonary disease, chronic obstructive):ti,ab,kw OR (chronic obstructive pulmonary disease*):ti,ab,kw OR (chronic obstructive airway disease):ti,ab,kw OR (chronic obstructive lung disease):ti,ab,kw OR (COAD):ti,ab,kw | 20,485 |
| #6 | (COPD):ti,ab,kw OR (chronic airflow obstruction*):ti,ab,kw OR (airflow obstruction, chronic):ti,ab,kw OR (airflow obstructions, chronic):ti,ab,kw | 20,402 |
| #7 | #5 OR #6 | 25,950 |
| #8 | #4 AND #7 | 82 |

## Scopus

| No. | Searches | Results |
| --- | --- | --- |
| #1 | TITLE-ABS-KEY(virtual reality) OR TITLE-ABS-KEY(VR) OR TITLE-ABS-KEY(virtual environment) OR TITLE-ABS-KEY(video game*) OR TITLE-ABS-KEY(virtual simulation) OR TITLE-ABS-KEY(virtual medicine) OR TITLE-ABS-KEY(mixed reality) OR TITLE-ABS-KEY(commercial game*) OR TITLE-ABS-KEY(virtual game*) OR TITLE-ABS-KEY(exergam*) OR TITLE-ABS-KEY(play-based therapy) OR TITLE-ABS-KEY(augmented reality) OR TITLE-ABS-KEY(virtual reality exposure therapy) OR TITLE-ABS-KEY(x-box 360) OR TITLE-ABS-KEY(kinect) OR TITLE-ABS-KEY(wii) OR TITLE-ABS-KEY(virtual world) OR TITLE-ABS-KEY(head-mounted display)) | 513,942 |
| #2 | (TITLE-ABS-KEY(pulmonary disease, chronic obstructive) OR TITLE-ABS-KEY(chronic obstructive pulmonary disease*) OR TITLE-ABS-KEY(chronic obstructive airway disease) OR TITLE-ABS-KEY(chronic obstructive lung disease) OR TITLE-ABS-KEY(COAD) OR TITLE-ABS-KEY(COPD) OR TITLE-ABS-KEY(chronic airflow obstruction*) OR TITLE-ABS-KEY(airflow obstruction, chronic) OR TITLE-ABS-KEY(airflow obstructions, chronic)) | 182,620 |
| #3 | #1 AND #2 | 295 |

## SinoMed

| No. | Searches | Results |
| --- | --- | --- |
| #1 | "虚拟现实"[常用字段] OR "VR"[常用字段] OR "体感互动"[常用字段] OR "3D眼镜"[常用字段] OR "VR眼镜"[常用字段] OR "头盔显示器"[常用字段] OR "头戴式显示器"[常用字段] OR "交互式情景模拟"[常用字段] OR "虚拟情景"[常用字段] OR "虚拟场景"[常用字段] | 9,005 |
| #2 | "慢性阻塞性肺疾病"[常用字段] OR "慢性阻塞性肺气肿"[常用字段] OR "COPD"[常用字段] OR "慢阻肺"[常用字段] OR "慢性阻塞性肺病"[常用字段] OR "慢性阻塞性气道疾病"[常用字段] OR "老慢支"[常用字段] | 85,347 |
| #3 | #1 AND #2 | 31 |

## VIP

| No. | Searches | Results |
| --- | --- | --- |
| #1 | U=( 虚拟现实 OR VR OR 体感互动 OR 3D眼镜 OR VR眼镜 OR 头盔显示器 OR 头戴式显示器 OR 交互式情景模拟OR 虚拟情景 OR 虚拟场景) AND U=(慢性阻塞性肺疾病 OR 慢性阻塞性肺气肿 OR COPD OR 慢阻肺 OR 慢性阻塞性肺病 OR 慢性阻塞性气道疾病 OR 老慢支) | 39 |

## WanFang

|  | Searches | Results |
| --- | --- | --- |
|  | (主题:(虚拟现实) or 主题:(VR) or 主题:(体感互动) or 主题:(3D眼镜) or 主题:(VR眼镜) or 主题:(头盔显示器) or 主题:(头戴式显示器) or 主题:(交互式情景模拟) or 主题:(虚拟情景) or 主题:(虚拟场景) ) and (主题:(慢性阻塞性肺疾病) or 主题:(慢性阻塞性肺气肿) or 主题:(COPD) or 主题:(慢阻肺) or 主题:(慢性阻塞性肺病) or 主题:(慢性阻塞性气道疾病) or 主题:(老慢支)) | 50 |

## CNKI

|  | Searches | Results |
| --- | --- | --- |
|  | SU='慢性阻塞性肺疾病'+'慢性阻塞性肺气肿'+'COPD'+'慢阻肺'+'慢性阻塞性肺病'+'慢性阻塞性气道疾病'+'老慢支' AND SU='虚拟现实'+'VR'+'体感互动'+'3D眼镜'+'VR眼镜'+'头盔显示器'+'头戴式显示器'+'交互式情景模拟' +'虚拟情景'+'虚拟场景' | 24 |
